# Understanding the Mechanisms that Promote Desistance from Sexual Offending: A Systematic Review

**DOI:** 10.1007/s10508-026-03484-4

**Published:** 2026-07-22

**Authors:** Tamara J. Smolinski, Gwenda M. Willis, Sarah M. Beggs Christofferson

**Affiliations:** 1 School of Psychology, Speech and Hearing, University of Canterbury, Private Bag 4800, Christchurch, 8140 New Zealand; 2 School of Psychology, University of Auckland, Auckland, New Zealand

**Keywords:** Desistance, Sexual offending, Systematic review, Criminality

## Abstract

Although the majority of individuals convicted of sexual offenses appear to desist from further sexual offending, theoretical and empirical efforts have traditionally concentrated on the mechanisms underpinning desistance from general criminal behavior. In recent years, however, scholarly attention has increasingly turned toward understanding the specific processes associated with desistance from sexual offending. Emerging evidence suggests that while general desistance theories offer partial explanatory value, they do not fully capture the processes unique to sexual offending desistance. To date, no systematic review has synthesized, using inductive methods, the existing literature to identify the mechanisms that facilitate desistance from sexual offending. Addressing this gap is both timely and essential. The present study undertook a systematic review aimed at identifying the mechanisms that promote desistance among adult men convicted of sexual offenses. A qualitative analysis of 29 empirical studies was conducted using thematic synthesis, resulting in the identification of eight distinct mechanisms. These mechanisms were categorized into two overarching groups: active and passive/avoidant. The groups seemed to differentiate between those who cease sexual offending and live a prosocial, adaptive life, and those who cease sexual offending but experience less prosocial change. The identified mechanisms demonstrate what may be necessary to overcome the barriers faced by people attempting to reintegrate into the community following a conviction for a sexual offense. To our knowledge, this is the first systematic review to empirically derive and propose potential mechanisms explaining how and why individuals desist from sexual offending.

**Supplementary Information:**

The online version contains supplementary material available at 10.1007/s10508-026-03484-4.

## Introduction

Although substantial research efforts have focused upon desistance from general and violent crime (Rocque, 2021), desistance from sexual offending remains comparatively less understood. Sexual offending research efforts have primarily concentrated upon sexual recidivism risk assessment (e.g., Hanson et al., 2007, 2015; Helmus et al., 2022; Wong et al., 2003), sexual recidivism risk factors (e.g., Mann et al., 2010; Seto et al., 2023), and the etiology of sexual offending (e.g., Marshall & Barbaree, 1990; Ward & Beech, 2006; Ward et al., 2005). In more recent times, a literature base has been established with a focus upon the protective factors that mitigate sexual recidivism risk, which has provided a strengths-based and balanced contribution to the field (de Vries Robbé et al., 2015; Kelley et al., 2022; Nolan et al., 2022; Willis et al., 2020). Yet these data continue to inform an approach focused on recidivism reduction rather than desistance promotion.

Mechanisms are utilized within science to provide an explanation for outcomes, by identifying the processes, entities, and activities through which change occurs (Illari & Williamson, 2012; Koch & Cratsley, 2020). A mechanisms-based approach to sexual offending desistance is timely. The cost of a reductionist approach to desistance research, which has focused on factors [of recidivism risk] and utilized dichotomous variables to inform desistance outcomes (i.e., recidivism), has been a loss of essential meaning about context and potential protective elements of relevant desistance processes (Fortune & Ward, 2017; Rocque, 2021; Ward & Fortune, 2016a, 2016b). Further, interventions that aim to reduce risk or enhance protective factors may fail to address the underlying mechanisms that lead to desistance (van den Berg et al., 2018), thereby reducing the effectiveness of said interventions. Currently, several theoretical proposals describe potential protective mechanisms for sexual offending desistance (de Vries Robbé, 2014; Thornton et al., 2017; Willis et al., 2021); however, empirical supporting evidence remains limited. To address this gap, the present study reviews and synthesizes qualitative and quantitative data from the current literature base, with the aim to identify protective mechanisms that promote desistance among adult men convicted of sexual offenses. 

### Conceptualizing Desistance

Historically, desistance was defined as the end of criminal activity and was typically measured by official data, such as the absence of arrest or reconviction (Glueck & Glueck, 1950; Shover, 1996). Modern definitions conceptualize desistance as a process that includes an interplay of cognitive, behavioral, and identity changes, setbacks, and progressions that occur within social contexts (Laws & Ward, 2011; Maruna, 2001; McNeill, 2015). As such, and consistent with prior sexual offending desistance research (Harris, 2017b), the current study was informed by the understanding that desistance is a complex, nonlinear, psychosocial process, and that sexual offending desistance is worthy of further research given that, for the most part, most individuals do desist (e.g., Hanson & Morton-Bourgon, 2005, 2009).

#### Methodological Challenges and Considerations in Desistance Research

Desistance research outcomes are often informed by data on individuals identified as nondesisters, namely those who have received a further official charge or reconviction (Maruna, 2001). Official reconviction data may underestimate reoffending, as nonoffending periods may reflect undetected crime and/or temporary gaps in offending (Carlsson, 2012; Kazemian, 2007; Liebling et al., 2023; Maruna, 2001; Ouellet, 2019). Further, law enforcement data are not exempt from subjective biases of the agencies and representative personnel who engage with persons convicted of offenses (Lauritsen & Cork, 2016; Sellin, 1931). This said, sexual recidivism data suggest that longer periods of offense-free community living increase the likelihood of desistance (Farmer et al., 2015; Hanson et al., 2014).

Triangulating official records with self-report data can improve the validity of recidivism outcomes (Krohn et al., 2010), as self-report research methodologies have resulted in the disclosure of officially undetected criminal behaviors (Abel et al., 1987; Lisak & Miller, 2002). Notably, self-reports are also subject to biases that may reduce their validity such as impression management (Tourangeau & Yan, 2007) or volunteer bias (McDonald et al., 2013; Rosnow & Rosenthal, 1976). Further, individuals neutralize, justify, and/or minimize their offending to protect their moral identities (Maruna & Copes, 2005; Sykes & Matza, 1957), which may further impact the validity of self-reports. However, narrative self-reports enable rich data and allow for the development of a complex understanding of the idiosyncratic nuances of desistance, such as exploring identity change and the internal transformations that occur along the desistance process (McAdams, 1985, 1993; Presser & Sandberg, 2015). As such, in recognition of the limitations of any single method (Gomes et al., 2018; Lauritsen & Cork, 2016; Sellin, 1931), this review will synthesize data from diverse methodologies.

### Desistance Theory

Desistance has traditionally been conceptualized through three broad processes: natural desistance/maturation, external social controls, and internal cognitive and identity shifts. General offending desistance research has well established that general offending typically declines with increasing age (Glueck & Glueck, 1950; Gottfredson & Hirschi, 1990; Moffitt, 1993). Although similar patterns also appear in sexual recidivism outcomes, some differences across sexual offense types are observed (Hanson, 2002; Hanson & Bussière, 1998; Hanson & Morton-Bourgon, 2005, 2009). Consequently, and to increase predictive accuracy, modern sexual recidivism tools include age adjustments that reflect the empirically established relationship between age and recidivism (Craig, 2008, 2011; Hanson & Morton-Bourgon, 2005; Helmus et al., 2012). As age is a static factor, a full review of the literature was considered beyond the scope of the current paper. Examination of external processes may therefore proffer more meaningful targets for supporting desistance.

External desistance processes include formal and informal social controls. Formal social controls include general and sexual-offense-specific criminal justice processes, sanctions, and policies. Sexual-offense-specific controls such as the Sex Offender Registration and Notification Act (SORNA; U.S. Department of Justice, 2025), place severe restrictions on individuals attempting to reintegrate after a sexual offense, with the overall aim to provide safer communities (Meloy, 2005; Paternoster, 2010). However, these legislative efforts have been shown to have no effect in reducing sexual recidivism (Call, 2018; Tewksbury & Jennings, 2010; Zgoba & Mitchell, 2023; Zgoba et al., 2018). Rather, sexual-offense-specific legislation obstructs reintegration by limiting access to housing, employment, and social support (Bailey & Sample, 2017; Lasher & McGrath, 2012; Tewksbury, 2005). Legislation intending to improve community safety thereby results in increasing stressors and paradoxically undermines desistance processes (Harris & Levenson, 2021; Levenson et al., 2016).

Sexual-offense-specific treatment, which sits at the intersection of formal social control and rehabilitation, has been associated with reduced recidivism (Gannon et al., 2019; Lösel & Schmucker, 2005; Schmucker & Lösel, 2015). Circles of Support and Accountability (Circles) is a sexual-offense-specific intervention aimed at reintegrating socially isolated individuals assessed at the highest risk of sexual recidivism (Azoulay et al., 2019). Circles demonstrated mixed and modest evidence of promoting sexual offending desistance (Clarke et al., 2017; Elliott et al., 2018). Notably, methodological variability between effectiveness studies of sexual-offense-specific formal controls may influence outcomes (Lösel & Schmucker, 2005; Lussier et al., 2023). Group-based approaches promote the development of prosocial processes such as empathy, prosocial identity, and cognitive change (Bartle, 2012; Harris, 2014, 2016; Höing et al., 2013; Kitson-Boyce et al., 2019), and the application of treatment learnings to reintegration (Beech et al., 1999).

Informal social controls (including family, employment, prosocial groups) have been shown to be strong predictors of general desistance (Laub & Sampson, 2001, 2003; Sampson & Laub, 1993, 2003, 2005). Yet the role of informal social controls in sexual offending remains complex and currently unclear due, in part, to such relationships potentially preceding or facilitating sexual offending (Cooley & Sample, 2018; Harris, 2014). Many individuals lose social support after a sexual offense conviction and go on to desist (Harris, 2017b). However, others maintain or renew their support networks, and credit informal support as central to their desistance (Bartle, 2012; Farmer et al., 2015). Further, informal social controls are often measured using dichotomous variables, which result in the loss of information as to the protective elements, or not, of these controls (Kruttschnitt et al., 2000; Lussier & McCuish, 2016). These findings highlight the need to better understand protective mechanisms that are specific to sexual offending desistance. Consequently, it may be that internal processes are most important for desistance given that stigma and structural barriers restrict access to external processes (Cooley, 2020; Harris, 2017b; Tewksbury, 2012).

Internal processes such as prosocial shifts in agency, identity, and cognition have been shown to be central to general offending desistance (Giordano et al., 2002; Maruna, 2001; Paternoster & Bushway, 2009). Although similar processes appear relevant in sexual offending (Farmer et al., 2012, 2015, 2016; Harris, 2017b; Hulley, 2016; Kras & Blasko, 2016; Milner, 2017), shame, stigma and restrictive policies tend to restrict the likelihood that internal shifts occur (Cooley, 2022; Harris & Levenson, 2021; Harris, 2017b). As such, interactionist theories may best explain the complex psychosocial processes that promote desistance (Bottoms et al., 2004; Farrall, 2005; LeBel et al., 2008; Serin & Lloyd, 2009). Specifically, the Integrative Theory of Desistance from Sexual Offending (ITDSO; Göbbels et al., 2012) provides a comprehensive theoretical conceptualization of the processes that are posited to promote sexual offending desistance.

The ITDSO (Göbbels et al., 2012) is a four-phase model informed by the Good Lives Model (GLM; Ward & Brown, 2004; Ward & Durrant, 2021); general desistance theories (Farrall & Calverley, 2006; Laub & Sampson, 2003; Maruna, 2001), transtheoretical behavior change (Prochaska & DiClemente, 1982), and the Risk Need and Responsivity framework (RNR; Bonta & Andrews, 2024). The first ITDSO phase, decisive momentum, involves internally or externally motivated decision-making that drives a person toward change when opportunities arise. Second, rehabilitation encompasses a shift towards the development of practical, prosocial identities and rebuilding the self. Third, *re-entry* highlights the need for sustaining motivation and prosocial behavior through barriers that arise during reintegration. The final phase, normalcy, is considered the point of true desistance and is achieved when individuals come to see themselves as non-criminal and are living prosocial meaningful lives. The ITDSO is conceptually and theoretically robust (Cohen, 2021; Lasher & McGrath, 2017), however the model lacks empirical support that the processes lead to desistance (Göbbels et al., 2014). As such, further research is needed to establish an empirically grounded understanding of the mechanisms that promote desistance from sexual offending.

### Study Aims

The aim of the current study was to identify potential protective mechanisms that promote desistance among adult men convicted of sexual offenses. Using thematic synthesis, the current study aimed to identify the underlying processes, entities, and activities that contribute to sustained desistance from sexual offending, expanding on existing theoretical and empirical frameworks.

## Method

The method and design for the current systematic review were guided by: the Cochrane Handbook for Systematic Reviews of Interventions (Higgins et al., 2022), the PRISMA statement (Liberati et al., 2009; Moher et al., 2015; Page et al., 2021a, 2021b), and the guidelines for PRISMA-protocols (PRISMA-P) (Page et al., 2021a, 2021b; Shamseer et al., 2015). The methodological protocol was registered in the International Prospective Register of Systematic Reviews (PROSPERO; registration number CRD42020166849).

### Eligibility Criteria

Report eligibility criteria were determined using the Population, Intervention, Comparator(s), Outcome, and Study Design framework (PICOS; McKenzie et al., 2022). The PICOS search tool was considered the most appropriate for the current review due to possible inclusion of multiple study designs, and published use in similar research (Methley et al., 2014). The inclusion and exclusion criteria are presented in Table 1.

**Table 1:** Systematic review inclusion and exclusion criteria

Inclusion Criteria	Description
Population	Biologically born males (18 years and older) who had been convicted of a sexual offense and were living in the community at the time the research was conducted
Intervention	The presence or addition of a specific prosocial and adaptive input or intervention such as sexual-offense relevant treatment, correctional service provision, or support system; or phenomena such as skill development, increased insight, or psychological changes
Comparator(s)	Matched groups, where possible. It is recognized that often, there are no control groups within sexual offending research due to the ethical dilemma of not providing an intervention. Consideration was given to non-matched groups during the quality assessment
Outcome	Desistance from sexual offending associated with the intervention/phenomena of interest. For quantitative studies, this was quantified in terms of recidivism outcomes attributed to the input. For qualitative studies, this was conceptualized as desistance from sexual offending attributed to the presence of the phenomena
Study Design	Quantitative, qualitative, and mixed-methods study designs written in the English language only. There were no time restrictions applied

Exclusion Criteria	Description
Treatment effectiveness studies	Treatment effectiveness studies were not included due to the collation of such results in previously conducted systematic reviews and meta-analyses which support the effectiveness of treatment for this population (e.g., Gannon et al., 2019; Lösel & Schmucker, 2005; Schmucker & Lösel, 2015)
Risk factors	Any studies that referred to desistance as a function of age or a reduction in risk factors only
Other populations	Any studies that included an adolescent or female population and those that explicitly considered specialist populations such as those with intellectual disability or forensic mental health
Other report types	Master’s theses, book chapters, conference proceedings, opinion pieces, meta-analyses, systematic reviews, and unstructured literature reviews
Non-English publications	Studies not published in English

### Search Strategy

Search terms were developed through multiple iterations to improve the specificity, sensitivity, and precision of the returned results. A broad search strategy was utilized (see Supplementary Material Table 1) as the benefits of appropriate references returned outweighed the extra screening burden. Searches were run by the first author in: PsycINFO (1887–present), Scopus (2004–present), Web of Science (1900–present), SocINDEX (1895–present), Embase (1947–present), and MEDLINE (1879–present) on January 27, 2020, and March 20, 2021; the second was limited to reports published after January 2020. A comprehensive search of gray literature was also completed.

### Information Sources

Peer-reviewed articles and unpublished doctoral theses were eligible and if both existed only the peer-reviewed article was included. Reports (a standalone research paper or thesis) were differentiated from studies (where the findings from one sample were reported across multiple publications; Lefebvre et al., 2022). The first author contacted twelve experts within the field to inquire about any unpublished research that may fit the inclusion criteria. In addition, the first author screened the reference list of the SAPROF-SO pilot version manual (Willis et al., 2017–2020) and all included articles, yielding four articles thus supporting the adequacy of the initial search strategy.

### Screening Process and Interrater Reliability

**Fig. 1:**
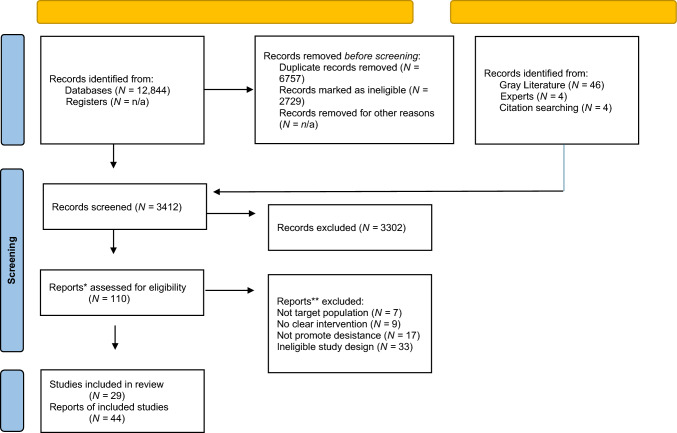
Flow diagram of article selection

The article selection process is shown in Fig. 1. All references were imported into EndNote and reports were screened using automated (keyword searches e.g., “female,” “adolescents” to exclude irrelevant titles in bulk) and manual approaches. The first author independently screened all reports, and a research assistant screened approximately 20% of the records to assess reliability (*n* = 208 titles/abstracts; *n* = 23 full texts). Any discrepancies were resolved through consensus, and the third author was available for consultation when needed.

Cohen’s (1960) kappa was chosen as the most appropriate means of assessing interrater reliability, and percentage agreement was calculated for comprehensiveness (Park & Kim, 2015). Title and abstract screening indicated substantial agreement (κ = 0.66; 83.8% exclusion agreement; 82.4% inclusion) and full-text screening indicated moderate agreement (κ = 0.46; 62.5% exclusion; 80.0% inclusion). Full-text screening discrepancies were primarily due to the inclusion of an incorrect design type, which when corrected resulted in 90.0% agreement. Final decisions for the remaining 10.0% were made by the first author. Full-text articles were imported into NVivo for qualitative review.

### Data Items

A standardized data form was developed by the first author using Excel, and data item headings were then created as file classifications and codes within NVivo to systematically extract the data. The first author developed a coding protocol to support systematic extraction of data items. File classifications included: reference type; study location, study type; sampling method; study year; total *n* (range); average follow-up period (years); indication of desistance; and screening outcome, definition of desistance, population characteristics (e.g., risk level, offense type, ethnicity); input characteristics (e.g., data source, duration, types, measures used); comparator characteristics (e.g., groups, attrition); outcome data; and study design and characteristics. Codes refer to the thematic labels given to the data during analysis, as elaborated on in the Data Synthesis section.

### Data Collection Process

The first author extracted all data items from all the included reports (*N* = 44), and the second reviewer extracted data items for *n* = 12 (27.3%) to examine the specificity of the coding protocol. The data items were cross-referenced, and any discrepancies were discussed for consensus. Before cross-reference, agreement was 95.5% across all file classifications and 85.0%–98.0% across all codes.

### Quality Assessment

A quality assessment tool was developed from the Critical Appraisal Skills Programme checklists (CASP; Critical Appraisal Skills Programme, 2019b). CASP was chosen for its accessibility and applicability across study designs, and documented use in systematic reviews (Alrashidi et al., 2023; Alzehr et al., 2022). Further, CASP was endorsed by Cochrane for synthesizing qualitative and mixed-methods evidence (Long et al., 2020). The quality assessment included nine items pertaining to Design, Methods, Results and Contribution, scored as “yes,” “no,” or “cannot tell.” As CASP did not prescribe a scoring system (Critical Appraisal Skills Programme, 2025), studies were considered poor quality if more than one “no” or “cannot tell” was assigned to any of the first six items related to design and methods. Disagreements between raters were resolved through consensus, with the availability of the third author if needed.

### Data Synthesis

Thematic synthesis (Thomas & Harden, 2008) was chosen as the most suitable analysis strategy, in line with Cochrane guidelines (Noyes et al., 2022). Thematic synthesis permitted secondary analysis of existing research and integration of both qualitative and quantitative findings (Fletcher et al., 2008; Lawrence & Willis, 2021). Thematic synthesis was preferred over deductive approaches such as framework synthesis (Dixon-Woods, 2011), particularly considering the limited research on protective mechanisms in sexual offending desistance.

Thematic synthesis involved a three-step process: (1) the first author conducted line-by-line coding of all results and discussion sections; (2) the first author identified descriptive themes within the data across all studies; and (3) all authors generated analytical themes that offered new explanatory insights from the data (Thomas & Harden, 2008). Analytical themes and their corresponding descriptive codes are presented in Supplementary Material Table 2. Reflexivity was maintained throughout the analysis, in recognition that the authors’ professional backgrounds and experiences may have influenced the interpretation of the data. Ongoing reflexive dialogue was used to critically examine positionality and enhance the credibility and trustworthiness of the findings. This stance acknowledged the provisional nature of the interpretations and the importance of transparency in qualitative synthesis.

**Table 2:** Descriptive characteristics of included reports

Report Characteristics					Sample Characteristics					Outcome		
Report	Country	Design			*N*	Offense Type			Intervention or Input	Desistance Indicator		*M* Follow up^b^
		Quan	Qual	Control		Child	Adult	Other		OR	SR	
Bartle (2012)^a^	New Zealand		✔		9	✔			Formal and informal social controls; internal desistance processes	✔	✔	49.0 (23–74)
Bates et al. (2012)	England	✔			60	✔	✔	✔	Circles	✔		36.2 (1–84)
Bates et al. (2014)	England	✔		✔	142	✔	✔	✔	Circles	✔		55.0 (4–114)
Bohmert et al. (2018)	USA		✔		10	✔			Circles	✔	✔	NR
Cesaroni (2001)	USA		✔		21	NR	NR	NR	Circles		✔	NR
Cooley (2020) ^a^	USA		✔		43	✔	✔	✔	Marriage, parenthood, employment		✔	NR
Cooley et al. (2017)	USA		✔		77	✔	✔	✔	Formal social controls	✔	✔	NR
Cooley and Sample (2018)	USA		✔		2	✔			Formal social controls	✔	✔	NR
Duwe and Donnay (2008)	USA	✔		✔	435	✔	✔	✔	Formal social controls	✔		96.0 (36–192)
Farmer et al. (2012)	England		✔	✔	10	✔	✔	✔	Dynamic desistance process		✔	NR
Farmer et al. (2015)	England & Wales		✔	✔	32	✔			Social context; Protective cognitions	✔	✔	60.0
Farmer et al. (2016)	England & Wales		✔	✔	32	✔			Protective cognitions	✔	✔	36.0
McAlinden et al. (2017)	England & Wales		✔	✔	32	✔			Informal social controls; Protective cognitions	✔	✔	60.0
Harris (2014)	USA		✔		21	✔	✔	✔	Natural desistance, cognitive transformation, informal controls		✔	48.0 (6–180)
Harris (2016)	USA		✔		60	✔	✔	✔	Individual desistance processes		✔	49.0 (4–252)
Harris (2017a)	USA		✔		45	✔	✔		Cognitive transformation		✔	24.0 (4–252)
Harris et al. (2017)	USA		✔		71	✔	✔	✔	Religion and spirituality		✔	51.6 (9–264)
Harris et al. (2019)	USA		✔		42	✔	✔		Pursuit/prioritization of Primary Human Goods		✔	24.0 (2–180)
Harris and Levenson (2021)	USA		✔		74	✔	✔	✔	Formal social control and reintegration		✔	49.0 (4–252)
Höing et al. (2013)	Netherlands & UK		✔		14	✔	✔	✔	Circles			NR
Höing et al. (2017)	Netherlands	✔	✔		17	✔	✔	✔	Circles		✔	12.0
Gulley (2016)	UK		✔		15	✔			Cognitive neutralizations in desistance		✔	78.0 (12–180)
Kewley et al. (2017)	Wales		✔		4	✔		✔	Religion		✔	NR
Kitson-Boyce et al. (2019)	England		✔		7	NR	NR	NR	Circles			NR
Kras (2021)	USA		✔		29	✔	✔	✔	Reintegration; Protective Cognitions	✔	✔	36.0
Kras & Blasko	USA		✔		28	✔	✔		Protective Cognitions	✔	✔	36.0
Kruttschnitt et al. (2000)	USA	✔			556	✔	✔		Formal and informal social controls	✔		60.0
Lytle et al. (2017)	USA		✔		25	✔	✔	✔	Romantic relationships	✔	✔	88.8
N. Mann et al. (2019)	England		✔		20	✔	✔		Formal social controls		✔	NR
Milner (2017) ^a^	England	✔		✔	47	✔	✔	✔	Early desistane processes; Circles	✔	✔	13.9; 16.3
Richards (2020)	USA & Canada		✔		31	✔	✔	✔	Circles		✔	58
Richards et al. (2020)	Australia		✔		11	✔			Cultural mentoring program	✔	✔	24.0 –108.0
Sample et al. (2018)	USA		✔		12	NR	NR	NR	Peer-to-peer mutual support group	✔	✔	NR
Scoones et al. (2012)	New Zealand	✔			196	✔			Release Planning	✔		132.1
Sowden & Olver (2017)	Canada	✔			185	✔	✔	✔	Treatment readiness and engagement (clinician-rated)	✔		111.6
Stansfield et al. (2019)	USA	✔		✔	2476	✔	✔		Humanist, spiritual, religious program involvement	✔		60
ten Bensel and Sample (2017)	USA		✔		112	✔	✔	✔	Formal social controls	✔	✔	99.6
ten Bensel and Sample (2019)	USA		✔		112	✔	✔	✔	Social media use	✔	✔	99.6
Walker et al. (2017)	Canada	✔			318	✔	✔		Family support	✔		36.0
Willis and Grace (2008)	New Zealand	✔		✔	81	✔			Community reintegration planning	✔		84.6;77.9
Willis and Grace (2009)	New Zealand	✔		✔	60	✔			Community reintegration planning			126.1; 126.0
Wilson et al. (2009)	Canada	✔		✔	88	NR	NR	NR	Circles	✔		35.8 (9–86); 38.6 (8–96)
Wilson et al. (2007)	Canada	✔		✔	120	✔	✔		Circles	✔		54.7 (3–123); 52.5 (3–124)
Woodward (2018)^a^	England & Wales		✔		10	✔			Reintegration and formal social controls		✔	8.0 (3–18)

^a^Doctoral Dissertation

^b^M months and/or Range months

Mean, SD = Standard Deviation, NR = Not Reported, OR = Official Recidivism Data; SR = Self-Reported Data

## Results

### Publication Selection and Study Characteristics

The review included 44 reports (40 peer-reviewed articles and four doctoral theses), representing 29 studies. Reports originated from Australia (*n* = 1), Canada (*n* = 4), the Netherlands (*n* = 2), New Zealand (*n* = 4), the UK (*n* = 12), and the US (*n* = 21). A total of 5,078 participants were included across all reports (*M* = 82.7, *SD* = 120.1, range = 4–476). Retrospective quantitative studies (*n* = 11) represented the most participants (*n* = 4,657; *M* = 423.4, *SD* = 698.8, range = 60–2,476); the qualitative studies (*n* = 16) included 422 participants (*M* = 26.4, *SD* = 28.3, range = 4–112) and two prospective mixed-methods studies recruited 64 participants (*M* = 32.0, *SD* = 21.2, range = 17–47). Studies utilized all-male samples, apart from two that reported a small percentage of their sample included females (0.5% and 3.0%, respectively). These studies were retained as female participants were excluded from risk assessment analyses.

Consistent with prior sexual offending research, most studies reported largely White European samples, although ethnicity was not quantitatively analyzed. Two studies reported upon desistance from sexual offending from Indigenous perspectives (Bartle, 2012; Richards et al., 2020). Although all studies described their samples as adults, ages were inconsistently reported, either not reported or where available ranged from 15 to 87 years. The two studies that included participants younger than 18 were not excluded given the inconsistencies in age reporting across the studies. Where available, mean ages were consistent with the sexual recidivism literature (approximately 30–50 years).

Desistance was measured as: 7 studies utilized self-reported desistance, 9 triangulated self-report with official records, 11 used official records only, and 2 did not clearly specify. Risk levels were inconsistently reported, and where provided, categories varied widely i.e., low to high. Report characteristics are presented in Table 2, and the aims and conclusions of all reports are summarized in Supplementary Material Table 3.

#### Quality Assessment of Included Reports

A quality assessment was conducted for all reports (*N* = 44). Overall, the designs, methods, and results of all reports were assessed to be clear and appropriate, and no reports were excluded based on methodological quality. Small sample sizes and study designs limited the generalizability of many studies, consistent with typical challenges cited within sexual offending and desistance research (e.g., Långström et al., 2013; Levenson & Prescott, 2014; Lösel et al., 2020; Marshall & Marshall, 2007). The full quality assessment procedures, interrater agreement statistics, and item-level ratings are reported in the Supplementary Material (Quality Assessment of Included Reports, Tables 4 and 5).

### General Overview: Protective Mechanisms

Eight mechanisms that may promote desistance from sexual offending were identified through thematic synthesis, categorized into two broad domains: active and passive/avoidant processes. The active mechanisms were: Prosocial Response to Formal Social Control, Positive Treatment Experiences, Development of Insight and Application of Understanding, Strengthened Personal Agency, Social Support and Accountability to Social Networks, and Living in Congruence with Values. Four processes were observed to be consistently present across the active mechanisms: sufficient internal resources, such as motivation, skills, and capacity; sufficient external resources, such as social support or stable housing; actions and observable steps taken by the individual; and supportive actions and contributions from prosocial others. The conceptualization of active mechanisms required the individual to enact their skills and engage with support, which differentiated them from the passive/avoidant mechanisms, where themes represented a lack of skill, capacity, and support.

The two passive/avoidant mechanisms were Desistance by Deterrence and Natural Desistance. These mechanisms represented individuals who had ceased offending but had yet to take proactive steps toward a changed life, nor did they report satisfaction or meaning in their lives. The distinction between the two groups aligns with the GLM’s (Ward & Brown, 2004; Ward & Durrant, 2021) emphasis on the contrast between surviving, characteristic of passive/avoidant mechanisms, and thriving, as seen in the active mechanisms.

The proposed mechanisms, informing studies, and illustrated examples of each mechanism are summarized in Supplementary Material Tables 6 and 7. The findings highlight that there is no universal pathway to desistance. For some individuals, active mechanisms emerged early and strengthened over time, whereas for others the active mechanisms developed more gradually. Some desisting individuals appeared to have active mechanisms that cooccurred with passive/avoidant processes, reflecting dynamic and individualized desistance pathways. Each mechanism is detailed below, with italics marking the mechanism and informing themes.

#### Prosocial Response to Formal Social Control

Prosocial Response to Formal Social Control was informed by two key processes: individuals’ exposure to formal controls (e.g., arrest, incarceration, community supervision) and their active, prosocial response to these experiences. For many, the experience of formal social control triggered a turning point for change (Bartle, 2012; Farmer et al., 2015; Harris, 2016; Harris & Levenson, 2021; Harris et al., 2019; Kruttschnitt et al., 2000; Milner, 2017). Desisting participants described cognitive shifts, including more positive attitudes toward supervision and professional support, and behavioral change, such as engaging with opportunities that became available to them.

#### Positive Treatment Experiences

Positive Treatment Experiences referred to the phenomena of treatment learning, interpersonal treatment dynamics, and the application of learning beyond the treatment setting, represented by four key themes. Many desisting individuals described treatment as a turning point for change, linked to shifts in their beliefs and attitudes about their offending (Bartle, 2012; Kras, 2021; Richards et al., 2020; Woodward, 2018). These effects were most evident among those with the internal and external resources that were required to accept responsibility for their offending and manage their subsequent shame. Presence of strengths-based and GLM-consistent approaches was associated with improved treatment experiences, and supported reintegration planning (Willis & Grace, 2008, 2009).

Second, peer support and mutual challenge within treatment groups facilitated self-reflection, personal growth, and access to nonjudgmental support (Bartle, 2012; Harris et al., 2019; Höing et al., 2013; Mann et al., 2019). Given the stigma associated with sexual offending, peers provided a rare sense of community (Kras, 2021). Skilled and respectful professionals further enabled safe group processes that encouraged support and challenge between group members.

Third, understanding my sexual offending process reflected the development of a personal narrative of change, and of prosocial motivations and behaviors for desisting individuals (Bartle, 2012; Cooley et al., 2017; Harris, 2017a; Mann et al., 2019). For those with adequate cognitive capacity and skills, developing a personal narrative supported coping with shame and enabled an integration of any new skills into daily life. GLM-informed and person-centered approaches were particularly effective in helping individuals identify their underlying needs and develop adaptive, non-offending strategies to have their needs met. Desistance was promoted when individuals perceived treatment as meaningfully contributing to their change, often well after program completion.

Finally, developing awareness of harm to others, including victims, was integral to some desistance narratives. Increased empathy signaled a motivational shift toward a good life and often represented a key turning point for change (Höing et al., 2017; Hulley, 2017; Kras, 2021; Milner, 2017). However therapeutic support and peer encouragement were critical in fostering empathy development, given the pain associated with recognizing the harm caused to others through their offending (Bartle, 2012; Cooley & Sample, 2018; Harris, 2014; Mann et al., 2019).

#### Development of Insight and Application of Understanding

Development of insight and application of understanding reflected active processes through which individuals developed insight and applied necessary skills, informed by four themes. Desisting participants were able to generalize and apply knowledge and skills across contexts. Early learning was primarily cognitive and involved trial and error as skills were practiced and refined. Over time, these skills became habitual components of daily functioning and their interpersonal interactions.

Self-regulation skills such as emotional regulation, problem-solving, and adaptive coping were identified as central to desistance from sexual offending. As deficits in self-regulation often precede sexual offending (e.g., Mann et al., 2010; Seto et al., 2023), developing an awareness of one’s regulatory state and responding appropriately were essential for desistance. Effective use of skills and receipt of constructive feedback appeared to strengthen one’s sense of control and confidence in managing interpersonal situations that arose. Improved problem-solving capacities further enabled access to social support and practical resources, promoting broader benefits including enhanced relationships and emotional and psychological well-being.

Developing and maintaining prosocial relationships were essential for accessing social support and were foundational to the six active mechanisms. Key interpersonal skills included perspective-taking, communication, empathy, and compassion. Desisting individuals demonstrated these skills through creating, repairing, and sustaining healthy relationships, that reinforced prosocial behavior.

Knowledge and skills related to safety enabled individuals to manage unhealthy sexual thoughts, urges, and behaviors. Desisting participants showed motivation and openness to discuss safety concerns, and applied plans as needed. They managed enduring offense-related interests by accepting their sexual thinking patterns and committing to cognitive and behavioral control. Desisting participants did not show complacency regarding their need to maintain safety for themselves and others. They used their skills to rebuild their lives and be active agents in their desistance.

Shame management emerged as a critical process in desistance. The means of managing shame varied, and the process of shame management appeared protective and motivating for many desisting individuals. Some participants confronted their shame directly, took responsibility for their actions, and, with adequate support, developed a more positive self-concept. Others used post hoc neutralizations such as attributing their sexual offending to contextual or situational factors that had since resolved. Neutralizations helped mitigate shame, maintain social support, and foster a prosocial identity. Attributing offending behavior to treatable or resolved issues was associated with increased confidence in one’s ability to change. Across studies, effective shame management differentiated desisting from non-desisting individuals (Bartle, 2012; Cooley, 2021; Farmer et al., 2015, 2016; Harris, 2016; Hulley, 2016; Kras et al., 2016; Milner, 2017; Richards et al., 2020) and was linked to navigating stigma and developing self-worth.

#### Strengthened Personal Agency

Strengthened personal agency reflected an individual’s capacity to act with motivation and intention, consistent with Bandura’s (1989) conceptualization of agency. Agency was supported by both internal resources (e.g., resilience, motivation) and external supports (e.g., prosocial networks) and was informed by five themes reflective of motivational, cognitive, and emotional processes that facilitated desistance. An internal locus of control represented an attributional style that was characterized by a perception of self-control over one’s life circumstances. An internal locus of control was central to developing and maintaining motivation which then led to decisive action, including supporting engagement in treatment, the continuation of safety through the management of unhealthy sexual thoughts, and the pursuit of meaningful prosocial goals such as obtaining employment and joining social groups.

Self-efficacy represented the belief in one’s ability to achieve success, and was common amongst desisting participants. Self-efficacy developed and was reinforced through applying learned skills, having the capacity for and engaging in self-reflection, and receiving constructive feedback from trusted others. Presence of self-efficacy was associated with resilience and perseverance through challenges. Self-efficacious individuals set meaningful and realistic goals that contributed to prosocial changes. A positive sense of self and a willingness to take risks further strengthened one’s capacity for self-efficacy, which in turn increased agency and personal growth.

Self-esteem and confidence reflected cognitive processes that appeared to develop through experiences of success and receipt of nonjudgmental feedback. Presence of self-esteem and confidence appeared to direct behavior, such as trying new activities. Self-reflection and feedback from others that their positive thinking led to desirable outcomes appeared to reinforce this cycle, promoting optimistic goals and actions aligned with desistance. Desisting individuals often had a positive mindset and attitude that enabled them to picture a productive and meaningful future. Having confidence that good things lay ahead helped to maintain motivation and prosocial behaviors. *Perseverance and resilience* encompassed the ability to continue working toward goals through knockbacks and challenges. A capacity for perseverance and resilience was observed amongst desisting participants, such as Harris’s (2016) resilient desisters, who demonstrated a sustained commitment to desistance. Perseverance and resilience were associated with a motivation and commitment to remaining active in one’s desistance process regardless of setbacks and challenges. Motivation, perseverance, and resilience appeared to breed prosocial action toward prosocial goals and desistance-promotive changes.

Finally, hope and optimism reflected emotional processes co-occurring alongside the aforementioned cognitive and motivational elements of Strengthened Personal Agency. Desisting participants expressed hope and optimism within their narratives, and the presence of these emotional processes appeared to play a critical role in maintaining agency. Quantitative findings demonstrated that hope and optimism supported desistance (e.g., Höing et al., 2013, 2017; Milner, 2017), enabling individuals to envision positive change and a better future. For some desisting participants, hope and optimism were strengthened as a function of experiencing prosocial identity changes and being able to imagine a prosocial future.

#### Social Support and Accountability to Social Networks

Social support and accountability to social networks highlighted the critical role of social support in promoting desistance for those who had access to it, as access to supportive networks distinguished active from passive/avoidant mechanisms. Four themes informed this mechanism: acquisition of support and three desistance-promotive processes that followed once support was established.

The acquisition of social support that promoted desistance came from many sources including family (Bartle, 2012; Bates et al., 2012; Cooley, 2020; Walker et al., 2017; Woodward, 2018), intimate partners (Bartle, 2012; Bates et al., 2012; Cooley, 2020; Lytle et al., 2017), friendships (Cooley & Sample, 2018; Richards, 2020; ten Bensel & Sample, 2017, 2019), and pastoral networks (Harris et al., 2017; Kewley et al., 2017; Lytle et al., 2017; Woodward, 2018). Desisting individuals reported high value in their relationships that had remained intact postconviction, which provided opportunities to apply their new interpersonal skills. Social support offered hope, connection, nonjudgmental acceptance, and alignment with personal values. Support also facilitated access to practical resources. However, findings suggested that desistance was determined by the following three processes, rather than the presence of any specific types of support.

Social support provided support and accountability. Prosocial others helped individuals manage risk, implement safety plans, and maintain overall safety. Desisting participants were open with trusted supports about their offense processes and relied on their networks to respectfully uphold their boundaries and provide accountability. These findings were consistent with informal social control processes observed in general desistance (e.g., Sampson & Laub, 1993, 2003), whereby family, friends, and community members reinforced social norms and contributed to sustained prosocial change (Bates et al., 2012; Bohmert et al., 2018; Cesaroni, 2001; Kitson-Boyce et al., 2019; Kruttschnitt et al., 2000; Wilson et al., 2009).

Prosocial roles such as involvement in family roles, involvement in friendships, participation in Circles (Höing et al., 2013, 2017; Kitson-Boyce et al., 2019; Milner, 2017), involvement in faith communities (Kewley et al., 2017), or involvement in peer support (Sample et al., 2018) enabled the contexts where purpose and belonging could develop. These roles provided a routine, reinforced self-worth, reduced loneliness, and encouraged prosocial behavior. Prosocial roles also offered opportunities to practice relationship skills and sustain meaningful connections, making the maintenance of established roles a priority for desisting individuals.

Prosocial connection referred to having supportive, trustworthy, and nonjudgmental others with whom individuals could speak openly. Such relationships were characterized by mutual respect and reciprocity and were particularly evident in the Circles model (Bohmert et al., 2018; Höing et al., 2013, 2017; Kitson-Boyce et al., 2019). Prosocial connection provided space for individuals to feel heard and valued, for mutuality, for sharing of challenges and successes, and for trust. As people convicted of sexual offenses often experience profound social isolation, regaining a sense of connection to others was especially important and supported the development of self-worth. Prosocial connection supported identity change toward becoming a contributing member of society. For example, “supportive preconviction relationships that persisted through conviction led to a sense of redemption for offenders that must navigate a new identity” (Lytle et al., 2017, p. 131).

#### Living in Congruence with Values

Living in Congruence with Values highlighted the importance of living in alignment with personal values to support and sustain desistance. Values motivated prosocial behavior and provided direction during reintegration. Several themes informed this mechanism. Desisting participants described living a meaningful life as one aligned with personal values and characterized by lifestyle stability, mental well-being, or purposeful activity (e.g., stable housing, simple lifestyles, entrepreneurship). Sexual offense convictions often led to profound loss, and the effort required to rebuild heightened participants’ appreciation for the positive change they had achieved. Maintaining valued gains appeared to reinforce ongoing prosocial behavior.

A sense of purpose was central to many desistance narratives, and was derived in many ways such as helping others, gaining employment, or being able to contribute socially. Engagement in valued activities further supported the development of self-worth, self-efficacy, and continued engagement in prosocial behavior. Generativity (e.g., Maruna, 2001), including mentoring or volunteer work, was frequently described as meaningful, as providing a sense of purpose, and as reinforcing positive change.

Stability, structure, and routine were foundational for consistent prosocial behavior. Desisting individuals maintained their lifestyle stability through realistic goal setting, goal reflection, engagement in purposeful activities, and engagement with prosocial others. When accessible, employment and training provided stability, although many participants faced postconviction barriers to obtaining meaningful work. Prosocial activities, including hobbies or social events, also facilitated lifestyle stability and social connection.

For some participants, particularly those who lacked support otherwise, the presence of spirituality and faith provided comfort, purpose, and social connection. Engagement in faith-based practices offered opportunities for routine, forgiveness, alignment with personal values, and the development of self-worth and hope.

Prosocial identity change was central to sustaining desistance. Participants described distancing themselves from a former self and adopting a prosocial identity consistent with general desistance theory (e.g., Giordano et al., 2002; Maruna, 2001; Paternoster & Bushway, 2009). Two subthemes captured this process. First, the Creation of a New Identity reflected cognitive transformation (e.g., Giordano et al., 2002), prosocial goals, adaptive behaviors, and increased self-esteem and self-worth (Harris, 2016; Höing et al., 2013; Hulley, 2016; Kras, 2021; Milner, 2017) that marked the creation of a new prosocial identity. Treatment, social support, and opportunities to demonstrate change were important facilitators for prosocial identity shifts. Richards et al. (2020) highlighted identity change as a collective process for Indigenous men, who grounded their prosocial identity in their ancestors, families, and cultures. "Men positioned their ancestors, families, and/or cultures (rather than individual selves) as unspoiled entities, constructing their true selves as inherently and indelibly connected to them" (Richards et al., 2020, p. 12).

Second, Separation from Sexual Offender Identity represented those individuals who distanced themselves from the “sex offender” label, often to cope with stigma and construct a nonoffending identity (Hulley, 2016; Sample et al., 2018; Woodward, 2018). Compared to identity creation, this process involved less openness and more strategic avoidance. Separation appeared adaptive when individuals lacked other resources for managing shame and stigma.

#### Desistance by Deterrence

Desistance by Deterrence represented a passive/avoidant mechanism in which offending had ceased, but change was driven primarily by aversive psychological states and avoidant coping. Individuals desisting through this process showed limited adaptive coping skills and tended to rely on avoidance, reflecting long-standing traits, reactions to arrest and conviction, or exacerbation by shame, stigma, and insufficient resources. Unlike active mechanisms, where avoidance served intentional risk management, individuals in this category avoided adaptive life contexts (e.g., prosocial groups, employment) and lived highly restricted lives.

Desistance by deterrence aligned with the concepts of "resignation" (Harris, 2016, p. 1726), "poor me/victim stance" (Milner, 2017, p. 170), and "traumatic coping" (Harris & Levenson, 2021, p. 776). Participants commonly exhibited rigidity, social isolation, negativity, and hypervigilance, often expressing hopelessness and continued identification with the “sex offender” label. Avoidance appeared to function as a means of managing shame and stigma in the absence of more adaptive strategies. Limited access to social support further distinguished this mechanism from active desistance pathways. Further, avoidance seemingly served multiple protective functions, including reducing potential exposure to further allegations, preventing unwanted disclosure of their offending histories, and responding to their fears of re-imprisonment.

#### Natural Desistance

Natural desistance represented the second passive/avoidant mechanism, characterized by the end of sexual offending, largely attributable to aging or the passage of time, rather than deliberate personal change or the influence of external intervention. Individuals desisting through this mechanism did not display evidence of desistance-oriented learning, such as insights gained from treatment. Instead, desistance was linked to factors such as advancing age, lifestyle changes, and reduced libido. Although passive and active elements may co-occur, offending cessation in these cases was primarily attributed by relevant participants to age-related processes. While recidivism rates reliably decline with age, it remains unclear whether this pattern reflects natural desistance alone or occurs alongside other psychological, social, or contextual influences.

## Discussion

The aim of the current review was to identify potential protective mechanisms that promote desistance from sexual offending. Through an inductive thematic synthesis of 29 studies, eight mechanisms were identified and conceptualized to be operating through either active or passive/avoidant processes. The six active mechanisms were Prosocial Response to Formal Social Control, Positive Treatment Experiences, Development of Insight and Application of Understanding, Strengthened Personal Agency, Social Support and Accountability to Social Networks, and Living in Congruence with Values. The two passive/avoidant mechanisms were: Desistance by Deterrence and Natural Desistance. Collectively, these findings offer an empirically informed conceptualization of desistance-promotive mechanisms that support a shift to consider these, from a predominant focus within the field on [risk and protective] factors and recidivism risk.

The thematic synthesis generated a conceptual framework for understanding potential desistance mechanisms that aligned broadly with several established desistance theories: the age-crime curve (Natural Desistance), deterrence/formal social controls (Prosocial Response to Formal Social Control, Positive Treatment Experiences, Desistance by Deterrence), internal desistance processes (Development of Insight and Application of Understanding, Strengthened Personal Agency, Living in Congruence with Values), and informal social controls (Social Supports and Accountability to Social Networks, Living in Congruence with Values). Although these findings represent an initial step toward identifying protective mechanisms, the resulting conceptual mappings enhance confidence in the empirical foundation of the review. Further, passive or avoidant mechanisms appear to be associated with primary (or act) desistance, the end of offending, whereas active mechanisms appear to align with secondary desistance, involving identity transformation and relational change in addition to offending cessation. The latter is widely regarded in the literature as essential for sustaining long-term behavioral change (Maruna & Farrall, 2003; Nugent & Schinkel, 2016). Notably, themes within the active mechanisms also reflected participants commencing their desistance through assisted desistance (Farrall, 2016; McNeill & Maruna, 2007), namely engagement in specific programs within prison to support their reintegration into the community.

The active mechanisms representing internal desistance processes (Development of Insight and Application of Understanding, Strengthened Personal Agency) and external desistance processes associated with social support (Social Supports and Accountability to Social Networks, Living in Congruence with Values) appeared to best align with all four ITDSO phases: decisive momentum, rehabilitation, re-entry and normalcy (Göbbels et al., 2012). These findings suggest that desistance is more likely to be facilitated when individuals possess sufficient internal capacity and have access to meaningful social support. As such, the finding that the passive/avoidant mechanisms were not consistent with the ITDSO is unsurprising. Mechanisms involving deterrence and formal controls appeared most relevant to the early ITDSO phases, whereas mechanisms reflective of internal processes characterize the transition toward normalcy, where adaptive functioning becomes internalized and habitual, rather than effortful. Further, Living in Congruence with Values aligned with identity-based explanations of desistance (Giordano et al., 2002; Maruna, 2001; Paternoster & Bushway, 2009), including processes of narrative change, prosocial identity development, and, for some, deliberate separation from stigmatizing labels (Evans & Cubellis, 2015; Spivey, 2024). Identity change was also a key component of the ITDSO. Although the mechanisms conceptually complement the ITDSO, as empirical support for the ITDSO remains limited (Göbbels et al., 2014; M. Cohen, 2021), further research is needed to examine whether the mechanisms identified here correspond to measurable desistance outcomes.

### From Protective Factors to Protective Mechanisms

The findings begin to elucidate the mechanisms by which protective factors may operate to promote desistance. Social Support and Accountability to Social Networks operated in the absence of specific protective factors such as intimate relationships or stable employment. The underlying processes of support and accountability, prosocial role engagement, and prosocial connection appeared central to desistance, aligning with social support desistance perspectives (Chouhy et al., 2020). Similarly, individual protective factors such as coping, self-control, and prosocial sexual interests were not sufficient in isolation. Desistance was determined by the application of skills within relevant contexts. Mechanisms such as Prosocial Response to Formal Social Control and Development of Insight and Application of Understanding required individuals to recognize when particular skills were needed and to apply them appropriately, rather than simply possessing the protective factor.

The case of empathy further illustrates this distinction. Developing the capacity for empathy, including victim empathy, was identified as a turning point for desistance within the review findings. Notably, meta-analytic evidence shows only weak associations between deficits in victim empathy and sexual recidivism (Hanson & Morton-Bourgon, 2005). However, qualitative research highlights the meaningful impact that victim-empathy work can have for individuals participating in treatment (Levenson et al., 2009, 2010; Wakeling et al., 2005). Although empathy as defined within protective factor assessment has been associated with reduced recidivism (Nolan et al., 2022), perhaps empathy may matter more for desistance-related narrative and identity processes than for predicting sexual recidivism. It is plausible that empathy facilitates, or is a by-product of, desistance when individuals possess the capacity for empathy, have opportunities to engage empathically with others, and can articulate their empathic understanding. Further research is necessary to clarify whether general or victim-specific empathy capacities are pertinent to desistance and to understand the mechanisms underpinning these processes.

Adaptive schemas, defined as prosocial global representations of the self, others, and the world, have been identified as a dynamic protective factor (Nolan et al., 2022; Willis et al., 2021). The review findings indicate that several active processes, including prosocial coping, self-esteem, engagement with prosocial others, and the capacity to develop a prosocial identity, may play a key role in fostering the development and activation of adaptive schemas. Notably, shame management enabled individuals to maintain a positive self-concept (Farmer et al., 2015, 2016), thereby aligning with broader human tendencies to rationalize past behavior as a means of separating oneself from mistakes. Thus, shame management strategies may have protective effects that contribute to forming and activating adaptive schemas, particularly among individuals whose desistance was facilitated by active, prosocial processes.

The findings suggest that it is the mechanisms that underlie protective factors that appear to facilitate desistance. An empirically sound understanding of the protective mechanisms that promote desistance from sexual offending offers potential advantages over the assessment of protective factors. First, mechanisms informed by empirical findings related to desistance may better inform therapy foci. Second, the assessment of protective factors may be best suited to recidivism risk assessment, whereas the assessment of mechanisms may more appropriately inform therapy and case management that aim to promote desistance. Further research is underway to explore these identified potential mechanisms in greater detail to ascertain their applicability within clinical practice.

### Presence of Passive/Avoidant Mechanisms

Not all desistance occurred through prosocial growth. Some people were simply surviving and not thriving in their attempt to desist from sexual offending, through avoidance, fear, and resignation. The GLM (Ward & Brown, 2004; Ward & Durrant, 2021) was developed to address the tension between a traditional risk management focus and a desistance focus that builds a meaningful, fulfilling life (Ward & Gannon, 2006). Desistance by deterrence does not therefore align with a GLM perspective. Researchers have used varying terms to describe this cohort of individuals, such as natural desisters (Harris, 2014), resigned desisters (Harris, 2016), traumatic coping (Harris & Levenson, 2021), and poor me/victim stance (Milner, 2017). It is also possible that the potentially nondesisters within Farmer et al.’s (2012, 2015, 2016) sample may have fit within this cohort. The passive/avoidant mechanisms were aligned with prior findings about the desistance-undermining impact of current sexual-offense-specific policies such as SORNA (Lasher & McGrath, 2012; Meloy et al., 2013; Tewksbury & Zgoba, 2010; Weinberger, 2019). The findings support that a move toward strengths-based and trauma-informed approaches to therapy and reintegration efforts will, at the very least, help address some of these difficulties (Levenson & Willis, 2019; Levenson et al., 2017).

It is possible that a lack of active mechanisms may represent future nondesisters, and the passive/avoidant mechanisms may identify a residual risk that could still be expressed through future recidivism. However, these individuals were shown by the respective studies to have achieved, at minimum, primary desistance (Maruna & Farrall, 2003). Perhaps individuals represented by the passive/avoidant mechanisms lack the capacity and support to ever reach secondary desistance (e.g., Maruna & Farrell, 2003) in the face of the structural disadvantage of attempting to reintegrate with a sexual-offense conviction.

### Implications

The current study provides a foundation for considering policy and practice from an empirically informed desistance perspective. Adopting a desistance-oriented approach to sexual offending intervention and rehabilitation could lead to more effective strategies that support successful reintegration rather than a primary emphasis on risk management, at the expense of desistance promotion. The GLM (Ward & Brown, 2004; Ward & Durrant, 2021) posits that offending occurs, in part, due to the presence of internal and external obstacles to meeting individual needs in prosocial ways. The GLM further states that by supporting individuals to increase their capacity and scope (i.e., the processes reflective across all active mechanisms) offending behavior will likely be reduced. Restrictive policies appear therefore counterproductive, as they undermine the processes necessary for active desistance, especially for those most in need of support. In contrast, policies that are informed by desistance research, are trauma-informed, and reduce structural barriers are more likely to contribute to the development of the mechanisms associated with desistance.

Future research is underway to develop a standardized coding protocol to evaluate the presence and strength of the identified mechanisms. Longitudinal studies could clarify whether the active mechanisms follow identifiable developmental sequelae, cluster in meaningful ways, and relate to desistance outcomes. Furthermore, targeted studies are needed with individuals desisting through passive/avoidant processes and with culturally diverse groups, both underrepresented in existing literature, to assess the generalizability of the findings and identify if any changes are required to the current conceptualization of the mechanisms.

The current findings also offer implications for clinical practice. Interventions should focus on strengthening the underlying active processes that facilitate desistance. For example, programs may benefit from scaffolding the transition from a cognitive understanding of treatment learnings to habitual skill use across contexts, supporting the development of meaningful prosocial roles and connections, or integrating meaningful values and identity work, consistent with the active mechanisms. Conversely, being able to assess for the presence of passive/avoidant mechanisms, particularly desistance by deterrence, may support individuals to shift from survival-oriented desistance toward more stable, agency-driven change. What appears essential is not the presence of a set number of mechanisms, but the presence of active processes to sustain desistance. At present, it remains unclear how many mechanisms may be necessary, or which mechanisms are most influential for desistance to be sustained; this work is also underway.

Although quantitative research will remain essential for validating potential relationships between the mechanisms and desistance outcomes, future work should move beyond a reliance upon dichotomous variables. Integrating narrative methods with quantitative models will be especially valuable, as qualitative data offer insights not captured by recidivism statistics alone. The mechanisms identified in this study thus serve as a theoretically grounded and empirically informed starting point for advancing mechanistic research on desistance from sexual offending.

### Limitations

To reiterate from the introduction, all research data, qualitative, quantitative, official or self-report, are subject to biases. Therefore, the use of self-narratives to inform this review, alongside quantitative findings, should not be regarded as a limitation. However, individuals’ perceptions of their own change processes are inherently subjective and may reflect idiosyncratic meaning-making rather than causal mechanisms of desistance (Laws, 2020). Further research is needed to determine whether the themes identified from self-narratives represent consistent and reliable mechanisms for change.

The review is also limited by the quality of the included studies. Many relied on qualitative, cross-sectional, or retrospective designs with small samples and few control groups, limiting causal inference and generalizability. Variability in methodologies and measurement tools makes it premature to draw firm conclusions about the relationship between proposed mechanisms and desistance. Although randomized controlled trials and prospective longitudinal designs would strengthen causal inference, they remain difficult to implement in this field (Långström et al., 2013; W. Marshall & L. Marshall, 2007). Given the emerging nature of sexual offending desistance literature, time is needed for high-quality longitudinal research to eventuate.

The search strategy for this review was intentionally broad. Although natural desistance emerged as one of the passive/avoidant mechanisms, this theme fell outside the study’s intended scope, as studies conceptualizing desistance solely as a function of age were explicitly excluded. Its presence in the synthesis reflects instances in which age-related processes were discussed within broader examinations of desistance, rather than studies focused exclusively on maturation. Consequently, natural desistance was captured only incidentally, and its representation in the current findings likely underestimates its prevalence in the wider literature. Future reviews employing alternative inclusion criteria may therefore identify a greater proportion of individuals desisting primarily through maturation-related processes. Relatedly, the strengths-based inclusion criteria used in this review may have unintentionally excluded studies examining risk constructs that also function as protective factors, given the conceptual overlap between risk reduction and strengths development (Serin et al., 2016). As a result, the mechanisms identified may differ from what would be observed under broader or distinct inclusion criteria. This should be considered when interpreting the findings and in designing future syntheses.

The review was informed by a narrow demographic profile, reflecting common constraints in desistance research. Recruitment processes likely introduced additional biases, given differences between individuals who participate in research and those who do not, as well as between those never caught, adjudicated, or otherwise involved in the criminal justice system (Harris, 2017a). Restrictions on treatment program participation for individuals who deny their offenses may also have excluded relevant desistance narratives, potentially underrepresenting passive and/or avoidant processes. Future research would benefit from larger, diverse samples and longitudinal designs to enhance reliability and assess the extent to which proposed mechanisms generalize across contexts. Finally, given the low base rate of sexual recidivism, examining desistance from all offending may provide a more comprehensive indicator of change (Lussier & Cale, 2013). Nonetheless, the current findings demonstrate that qualitative self-report data offer rich contextual insights not captured through recidivism outcomes alone. Further exploration of these issues is warranted.

### Conclusion

Given the significant societal costs of sexual crime, advancing research on desistance is essential. The current systematic review identified eight mechanisms that may promote desistance from sexual offending through an inductive thematic synthesis. The identified mechanisms align with established desistance theories and offer a theoretically coherent and empirically grounded foundation. The mechanisms are not posited as definitive causal explanations, as establishing causation is inherently more difficult than identifying associations. Certainty in the mechanisms is not possible without strong empirical and theoretical support (Ward, 2019). Accordingly, the mechanisms proposed here should be viewed as tentative. Further work is required to validate these proposed mechanisms and determine their relevance to desistance. The present findings provide a foundation for future research and highlight potential mechanisms that, if supported, may inform interventions to promote desistance from sexual offending.

## Supplementary Information

Below is the link to the electronic supplementary material.

Supplementary file1 (DOCX 68 kb)

## Data Availability

The methodological protocol for this study was submitted for pre-registration in the International Prospective Register of Systematic Reviews (PROSPERO) database (registration number CRD42020166849).
