# Supplementary material for: Understanding the Mechanisms that Promote Desistance from Sexual Offending: A Systematic Review
Source: Arch Sex Behav. 2026 Jul 22;55(5):1877–98. doi: 10.1007/s10508-026-03484-4 (PMC13427852; doi:10.1007/s10508-026-03484-4)
Supplement: Supplementary file 1 — Supplementary file1 (DOCX 68 kb) [file 10508_2026_3484_MOESM1_ESM.docx]

Supplementary Material Table 1

*Final Search Terms and Associated Syntax*

| Concept | Syntax |
| --- | --- |
| Sexual offending | DE "Sex Offenses" OR DE "Sexual Abuse" OR DE "Incest" OR DE "Rape" OR DE "Pedophilia" OR "sex* abus*" OR "sex* violen*" OR "sex crim*" OR "sex* offen*" OR rape OR incest* OR pedophil* OR paedophil* |
| Recidivism | (DE "Recidivism") OR (reoffen*OR recidivis*) |
| Desistance | “Desist*” |
| Protective factors | “Protec* factor*” |
| Population | male or men or man or males |

**Supplementary Material** **Table 2**

*Broad Level and Informing Codes*

| Broad Level Codes  (Analytical themes) | Informing Codes  (Descriptive themes) | |
| --- | --- | --- |
| Prosocial response to formal social control | Exposure to formal social controls | Experience of any formal social control (police, criminal justice system, prison, probation, sexual offence specific legislation) |
|  | Prosocial response | Formal social control as the starting point for motivation to desist  Making the most of opportunities that arose  Engagement through challenge |
| Positive treatment experiences | Turning point for change | Accepting and taking responsibility  Shifting cognitions  Positive group dynamics and committed group members  Skilled, warm, and respectful professionals  Approach goals  GLM aligned |
|  | Peer support and mutual challenge | Active involvement in groups  Learning from others which encourages own thinking  Sense of community |
|  | Understanding my sexual offense process | Being vigilant about problem behaviors  Personal story of change – idiosyncratic  Understanding how and why  Knowing what needs to change |
|  | Awareness of harm caused to others | General empathy development  Victim empathy development |
| Development of insight and application of understanding | Self-regulation | Emotional regulation  Problem-solving  Adaptive coping  Self-reflection |
|  | Relationships | Perspective-taking  Communication  Compassion  Empathy |
|  | Safety | Manage unhealthy sexual thoughts, urges, and behaviors  Discuss safety with support people  Apply safety plans |
|  | Shame management | Shame as motivating and protective  Post hoc neutralizations  Situational or contextual explanations for sexual offending |
| Strengthened personal agency | Internal locus of control | Feeling in control of their future, actions, cognitions  Desistance as a personal choice |
|  | Self-efficacy | Positive cognitions and self-talk  Self-belief  Recognizing own strength and ability  Working towards goals |
|  | Self-esteem and confidence | Trying new things, experiencing success  Positive mindset  Seeing a positive future  Positive feedback from others |
|  | Perseverance and resilience | Persistence through setbacks and challenges  Committed to long term change  Keeping at it |
|  | Hope and optimism | Holding on to hope  Seeing a positive future |
| Social support and accountability to social networks | Acquired social support | Family  Friendships  Intimate relationships  Pastoral support  Access to practical resources |
|  | Resultant processes | Support and accountability  Prosocial connection  Prosocial roles |
| Living in congruence with values | Living a meaningful life | Values aligned behavior  Making and sustaining change |
|  | Sense of purpose | Generativity and giving back  Involvement in values driven social activity |
|  | Stability | Stability, keeping busy, structure and routine  Goal setting, purposeful activities, engagement with prosocial others  Prosocial activities |
|  | Spirituality and faith | Faith-based practices  Forgiveness  Alignment with personal values  Development of self-worth and hope |
|  | Prosocial identity | Creation of a new identity  Separation from “sex offender” label |
| Desistance by deterrence | Avoidance to cope with reintegration  Aversive psychological states  Avoidant coping | Avoid allegation of future sexual offences  Avoid negative impacts on their support  Dealing with stigma  Fear of returning to prison |
|  | Unable to separate self from “sex offender” label | Continuous identification with labels  Hopelessness  Limited supports and prosocial feedback |
| Natural desistance | Age as a natural desistance mechanism | Offending stopping due to growing older or lifestyle changing |
|  | Desistance without input | Stopped offending on my own  Treatment didn’t help me |

Supplementary Material Table 3
*Primary Aims and Key Conclusions of Included Reports*

| Report | Report Type | Primary Aims | Key Conclusions |
| --- | --- | --- | --- |
| Bartle (2012) | Thesis | To investigate the role of treatment in understanding what motivated and supported desistance. | Formal and informal social controls were mutually motivating and undermining desistance processes, impacting the narratives participants created around their offending and their desistance. |
| Bates et al. (2012) | Journal Article | To examine how the needs of sixty core members were met and how Circles help to support their desistance. | Circles supported the core members' reintegration whilst upholding community safety. |
| Bates et al. (2014) | Journal Article | To describe the first 10 years of the implementation of Circles in South-East England. | Circles supported individuals assessed as having a high risk of recidivism to safely reintegrate into the community after imprisonment for a sexual offence. |
| Bohmert et al. (2018) | Journal Article | To describe the needs of core members and the types of support provided to them. | Core members required various social support depending on their individual needs. Overall, core members were satisfied with the support they received, and some notable benefits were practical assistance, emotional support, and friendship. |
| Cesaroni (2001) | Journal Article | To explore whether Circle's goals of supportive reintegration and community accountability are contradictory and how core members and volunteers work together. | Support and accountability goals were able to operate concurrently, and core members reported that Circles supported their safe reintegration into the community. Volunteers were motivated to work with Circles to improve community safety. |
| Cooley (2020) | Thesis | To examine using qualitative analysis how the institutions of marriage, parenthood, and employment facilitated sexual offending or supported desistance. | Institutions (informal social controls) were observed to facilitate and inhibit sexual offending. Findings show that it is imperative to accurately capture how institutions change over time as their influence on desistance is dynamic. |
| Cooley et al., (2017) | Journal Article | To explore how formal social controls act as deterrents for individuals convicted of sexual offences. | Deterrence effects, such as the threat of legal sanctions for rule violations, were shown to work to support desistance in this sample. |
| Cooley & Sample (2018) | Journal Article | To demonstrate using a comparative case study, that desistance is more than a statistical lack of reoffending. | Desistance involved behavioral, emotional, and identity changes not necessarily required for an empirical reduction in recidivism. |
| Duwe & Donnay (2008) | Journal Article | To examine if community notification reduces sexual reoffending. | Evidence suggested that community notification had a deterrent effect on sexual recidivism for the utilized sample. |
| Farmer et al. (2012) | Journal Article | To investigate the processes by which desistance from sexual offending occurs, compared between desisting and nondesisting participants. | Different processes were observed between desisting and nondesisting participants, conceptualized through the primary human goods. Desistance was associated with communion and agency. Further, the desisters accepted responsibility for their offending and were optimistic. Treatment was identified as a key turning point for desistance. |
| Farmer et al. (2015) | Journal Article | To develop a theoretical and empirical understanding of desistance from sexual offending. | Desisters used post hoc neutralizations attributing their offending to situations or contexts since resolved. They framed their desistance as in their control and were optimistic about their future. Arrest and treatment were identified as turning points for change. |
| Farmer et al. (2016) | Journal Article | To explore the role of situational themes in the narratives of individuals who desist from sexual offending against children. | Framing sexual offending as something that occurred due to situations or contexts that were no longer relevant or resolved was considered a form of shame management that supported changes to a prosocial identity, thus supporting desistance. |
| Harris (2014) | Journal Article | To explore the extent to which theories of desistance from nonsexual offending explain desistance from sexual offending. | Findings found mixed support for established theories of desistance. Themes consistent with cognitive transformation and natural desistance were observed, and more positive themes were associated with cognitive transformation. |
| Harris (2016) | Journal Article | To explore desistance from sexual offending by attending to the way the men themselves described their understanding and experience of their desistance. | Four styles of desistance were identified. Support was found for theories citing natural desistance and aging out (retirement). The remaining three styles (resignation, rote, and resilience) shared little similarities to established general desistance theories that emphasize the role of informal social controls and cognitive transformations. |
| Harris (2017a) | Journal Article | To describe and explain desistance from sexual offending. | Desistance from sexual offending appears to occur without the achievement of primary human goods, in the absence of informal social controls, and/or other processes described in extant desistance literature. The findings underscored that desistance from sexual offending requires its own literature. |
| Harris et al. (2017) | Journal Article | To examine the role of religion and spirituality in desistance from sexual offending. | Religion and spiritualism offered faith, positivity, emotional regulation/religious coping, forgiveness/ redemption, social bonds/connectedness, and routine/ritual. These processes facilitated the development of self-control and social capital. |
| Harris et al. (2019) | Journal Article | To examine the extent to which each man discussed desiring, pursuing, and attaining the specific primary human goods outlined in the GLM. | The primary human goods of life and survival, relationships and friendships, and knowledge were observed within participants' narratives; however, only knowledge was achieved by many participants. Many barriers were observed in pursuing primary human goods, and treatment was reported to be instrumental in gaining primary and secondary human goods. |
| Harris & Levenson (2021) | Journal Article | To examine through a trauma lens the impact that formal social controls and stigma have on individuals attempting to reintegrate following a sexual offence conviction. | Participants primarily coped by hypervigilance, reliance on the insurance of surveillance, and fear and avoidance goals. |
| Höing et al. (2013) | Journal Article | To examine how and why Circles can be effective in the prevention of sexual recidivism for individuals assessed as medium and high risk. | Circles supported the development of a positive narrative self and the improvement of social and human capital. Circles facilitated hope, self-esteem, and sustained motivation to change. Further, Circles addressed risk-related attitudes and behavior, thereby supporting re-entry and desistance. |
| Höing et al. (2017) | Journal Article | To further understand desistance processes in core members and to explore the contribution of Circles by using a prospective design. | Circles supported core members to improve their agency, self-regulation, and self-reflection skills. Circle discussions facilitated these processes but only when embedded in a context of trusting and reciprocal Circle relationships. |
| Hulley (2016) | Journal Article | To explore the presence and role of neutralization for fifteen men self-reporting desistance from further sexual offending. | Findings showed that neutralization were necessary early in desistance and that treatment was a turning point for adaptive changes. However, neutralization ceased with the passage of time and the change to a prosocial identity. These processes were dependent upon access to social and structural support. |
| Kewley et al. (2017) | Journal Article | To examine the role of affiliation to a religious community in supporting reintegration back into the community following a conviction for sexual offending. | Engagement with a religious or spiritual community enhanced reintegration for those religiously inclined. However, the psychological transition from “offender” to “non-offender” was complex, even with support from a religious community. |
| Kitson-Boyce et al. (2019) | Journal Article | To learn from core member and volunteer experiences from being involved in a Circle that transitions from prison to community. | Volunteers provided core members support and accountability. Further, they encouraged the development of support networks outside of the Circle to support their transition should the Circle close. |
| Kras (2021) | Journal Article | To explore the patterns of desistance among men convicted of sexual offences and how the men express cognitive scripts, considering the influence of treatment and social stigma. | Evidence was found for Maruna’s (2001) redemption and condemnation scripts. However, these findings were contextualized by labelling and treatment effects. Redemption scripts reflected desistance and a primary mechanism for rejecting the sex offender label. Condemnation scripts reflected the universal experiences of stigma. |
| Kras & Blasko (2016) | Journal Article | To consider how post hoc accounts of offending behavior provided by men convicted of sexual offences related to their desistance processes. | Desisters expressed post hoc explanations for their sexual offending that reflected a combination of responsibility-taking and externalization of responsibility to a situation now resolved. Authors considered that perhaps situation post hoc explanations support a transition to a noncriminal identity. |
| Kruttschnitt, et al. (2000) | Journal Article | To explore what role formal and informal controls have in desistance from sexual crime. | Desistance was measured as the absence of official reoffence, and most participants who experienced the combined effects of formal and informal social controls were particularly likely to desist. |
| Lytle et al. (2017) | Journal Article | To explore how the quality of romantic relationships supports desistance from sexual offending. | The nature of the support from romantic relationships depended upon the relationship's timing. Relationships that continued from preconviction led to a sense of redemption that individuals could navigate to a new prosocial identity. Relationships that commenced postconviction provided support to manage stigma, the criminal justice system, support to maintain a positive self-image and a chance to experience better relationships. All relationships offered uniformity and acceptance. |
| N. Mann et al. (2019) | Journal Article | To understand how individuals convicted of sexual offences navigate the barriers brought about by formal and informal control mechanisms from sexual offence-specific legislation and the impact of these on desistance processes. | When a " welfare " and personal approach was adopted, participants felt able to access support and make desistance-oriented interpersonal changes. Participants who received a “surveillance” approach found this to undermine their opportunities for support, such as access to family, which would have otherwise supported their desistance. |
| McAlinden et al. (2017) | Journal Article | To understand the role that informal social controls (employment and relationships) and cognitive changes (seeing future selves) have in supporting desistance from sexual offending. | Identity changes were evident in participants who were desisting. Relationships and work were perceived as key to the development of a new future self. |
| Milner (2017) | Thesis | To explore the early stages of desistance from sexual offending (within the first two years), how individuals manage their sexual interests, and consider the relevance of extant desistance theory with a particular focus on identity change. | The early stages of desistance were broadly encapsulated into three groups that represented low, medium and high scores on measures related to hope, social connectedness, and locus of control. Processes identified as most important for desistance were high levels of hope, optimism and an internal locus of control. |
| Richards (2020) | Journal Article | To examine participants’ understandings of their desistance from sexual offending and what role Circles had in this. | The social relations that result from Circles were essential to their operation and effectiveness. The social relations formed within circles shape action and support the production of desisting identities. |
| Richards et al. (2020) | Journal Article | To examine how the cultural mentoring program supported self-narratives and identity shifts to a future law-abiding self. | Redemption scripts were evident in the men's narratives; they reflected their cultural true selves and cultural goals. Narratives ascribed external and collective forces as impacting desistance rather than presenting with an individualistic lens. |
| Sample et al. (2018) | Journal Article | To understand how a peer-to-peer social support group operates to support registered individuals and their families, navigate stigma and labelling effects, maintain well-being and contribute to society. | Fearless provides a safe and nonjudgmental space to share, discuss difficult topics, seek support and develop friendships. Several quality-of-life indicators (friendships, employment, self-confidence) increased, and stress and isolation decreased. |
| Scoones et al. (2012) | Journal Article | To examine the role of release planning in supporting reduced recidivism (alongside the relative contributions of static and dynamic risk) for individuals convicted of sexual offences. | Assessment of release planning increased predictive accuracy for recidivism beyond static and dynamic risk measures. |
| Sowden & Olver (2017) | Journal Article | To examine the role of clinician-rated client treatment variables in treatment retention and recidivism for individuals seeking sexual offence-specific treatment. | Treatment completion was associated with engagement in treatment and “constructive” treatment behavior, regardless of assessed risk or criminogenic need. |
| Stansfield et al. (2019) | Journal Article | To examine whether participation in humanist, spiritual and religious services, religious orientation, and problem-solving style is associated with reduced recidivism. | Participation in religious or spiritual services may support reintegration and reduce recidivism. Engagement with humanist, spiritual, and religious services provided social support and connection with others who see their worth outside of their offence. |
| ten Bensel, & Sample (2017) | Journal Article | To understand the impact that public sex offender registries have on social connections and serve as a way for registered individuals to seek support. | All 112 participants subject to public registers reported a sense of collective identity. They could share and feel a sense of community and connectedness, thus creating a sense of social belonging. |
| ten Bensel & Sample (2019) | Journal Article | To explore how individuals subject to the public “sex offender” register and their families cope with social isolation and how the Internet can support social connectedness | Internet and social media use helped to reduce some participants' feelings of isolation and worthlessness. Four broad categories were determined, ranging from isolated individuals with little to no social capital or Internet use to active users of the Internet and social media for social connection and the development of social capital. |
| Walker et al. (2017) | Journal Article | To explore the role of family support on offending behavior and desistance from sexual offending. | Desistance (reduced recidivism) was associated with stronger family support. |
| Willis & Grace (2008) | Journal Article | To determine the role of community reintegration planning in supporting reductions in sexual recidivism. | Better-quality release planning was associated with reduced recidivism, especially in the areas of accommodation, employment, and GLM secondary goods. |
| Willis & Grace (2009) | Journal Article | To determine the role of community reintegration planning in supporting reductions in sexual recidivism. | Findings supported prior research that better quality release planning was associated with reduced recidivism. Planning for accommodation, employment, and social support resulted in the best predictive model for sexual recidivism. |
| Wilson et al. (2009) | Journal Article | To evaluate whether Circles is an effective approach to support the reintegration of individuals assessed as high risk of sexual recidivism. | Engagement with Circles was associated with reduced rates of reoffending compared with individuals matched on risk who did not engage with a Circle. |
| Wilson et al. (2007) | Journal Article | To evaluate the effects of Circle to support the reintegration of individuals convicted of a sexual offence. | Circles were associated with a 70% reduction in sexual recidivism compared to that of the matched comparison sample. Observed rates were less than one-quarter of the predicted actuarial sexual recidivism rates. |
| Woodward (2018) | Thesis | To explore the desistance processes of individuals who have committed child sexual offences, particularly related to social processes, risk management and stigma. | A range of social processes was observed regarding how connected participants were with others, how they navigated sexual offence-specific conditions, and their willingness to risk their privacy and confidentiality when disclosing to others to negotiate stigma. Agency supported reintegration. |

Supplementary Material – Quality Assessment of Included Reports

An assessment was completed of the quality of each report (*N* = 44) included in the review. The consistent use of the quality assessment tool across the two raters for *n =* 10 reports was calculated as a percentage agreement, with an average agreement of 90.0% across all questions (see Supplementary Material Table 4). The lowest agreement was 60.0% for question eight. Upon closer inspection, disagreements were observed for *n* = 5 reports between the middle anchor ‘cannot tell’ and either ‘yes’ or ‘no’ (i.e., there were no instances where one rater gave a ‘yes’ and the other ‘no’). As such, the lower agreement for this item was not considered problematic, and a consensus was reached through discussion.

Overall, the design, methods, and results for all included reports were clear and appropriate, as reported in Supplementary Material, Table 5. As all reports were given a ‘yes’ for the design and method questions, other than *n* = 9 that were given a ‘cannot tell’ for question four, none were excluded. For these *n* = 9 reports, it was unclear whether the authors had considered and accounted for bias within their respective study designs. The primary concern highlighted from the assessment was whether the results were considered generalizable. For question eight, *n* = 12, twelve reports were rated as ‘cannot tell’, and *n* = 18 were rated as a ‘no’. Primarily, these ratings were given due to small sample sizes or study design (i.e. qualitative or retrospective without a control group), consistent with known methodological limitations in sexual reoffending research (e.g., Långström et al., 2013; Levenson & Prescott, 2014; Lösel et al., 2020; Marshall & Marshall, 2007). These findings reflected the nonlinear and complex process of desistance and challenges to desistance research; all studies were considered to contribute to the field despite their limitations. Further, the underrepresentation of longitudinal study designs was unsurprising given that research on desistance from sexual offending has only gained significant attention in the past decade.

Table 4

*Quality Assessment Tool Percentage Agreement Across Raters for All Items*

| **Category** | **Questions** | **Percentage Agreement*** |
| --- | --- | --- |
| Design | Are the aims or research questions clearly defined? | 100% |
|  | Is the chosen design appropriate? | 100% |
| Methods | Has bias been considered and accounted for? | 90% |
|  | Is the recruitment strategy appropriate? | 90% |
|  | Are the chosen methods appropriate? | 100% |
| Results | Are the results clearly reported? | 90% |
| Contribution | Do the benefits of the research outweigh any costs? | 80% |
|  | Are the results generalizable? | 60% |
|  | Do the results add helpful evidence to the field? | 100% |

*Note.* Adapted from Critical Appraisal Skills Programme (2019a, 2019c, 2019d, 2019e)

Supplementary Material Table 5
*Quality Analysis of Included Reports*

|  | Design | | Method | | | Results | Offers a contribution | | |
| --- | --- | --- | --- | --- | --- | --- | --- | --- | --- |
| Report | Are the aims or research questions clearly defined | Is the chosen design appropriate | Is the recruitment strategy appropriate | Has bias been considered and accounted for | Are chosen methods appropriate | Are the results clearly reported | Do the benefits of the research outweigh any costs | Are the results generalizable | Do the results add helpful evidence to the field |
| Bartle (2012) | Yes | Yes | Yes | Yes | Yes | Yes | Yes | No | Yes |
| Bates et al. (2012) | Yes | Yes | Yes | Cannot tell | Yes | Yes | Yes | No | Yes |
| Bates et al. (2014) | Yes | Yes | Yes | Yes | Yes | Yes | Yes | Cannot tell | Yes |
| Bohmert et al. (2018) | Yes | Yes | Yes | Cannot tell | Yes | Yes | Yes | Yes | Yes |
| Cesaroni (2001) | Yes | Yes | Yes | Yes | Yes | Yes | Yes | Cannot tell | Yes |
| Cooley (2021) | Yes | Yes | Yes | Yes | Yes | Yes | Yes | No | Yes |
| Cooley et al., (2017) | Yes | Yes | Yes | Yes | Yes | Yes | Yes | No | Yes |
| Cooley & Sample (2018) | Yes | Yes | Yes | Yes | Yes | Yes | Yes | No | Yes |
| Duwe & Donnay (2008) | Yes | Yes | Yes | Yes | Yes | Yes | Yes | Cannot tell | Yes |
| Farmer et al. (2012) | Yes | Yes | Yes | Yes | Yes | Yes | Yes | Cannot tell | Yes |
| Farmer et al. (2015) | Yes | Yes | Yes | Yes | Yes | Yes | Yes | No | Yes |
| Farmer et al. (2016) | Yes | Yes | Yes | Cannot tell | Yes | Yes | Yes | Yes | Yes |
| Harris (2014) | Yes | Yes | Yes | Yes | Yes | Yes | Yes | Yes | Yes |
| Harris (2016) | Yes | Yes | Yes | Yes | Yes | Yes | Yes | No | Yes |
| Harris (2017) | Yes | Yes | Yes | Yes | Yes | Yes | Yes | Yes | Yes |
| Harris et al. (2017) | Yes | Yes | Yes | Yes | Yes | Yes | Yes | No | Yes |
| Harris et al. (2019) | Yes | Yes | Yes | Yes | Yes | Yes | Yes | No | Yes |
| Harris & Levenson (2021) | Yes | Yes | Yes | Yes | Yes | Yes | Yes | No | Yes |
| Höing et al. (2013) | Yes | Yes | Yes | Yes | Yes | Yes | Yes | Cannot tell | Yes |
| Höing et al. (2017) | Yes | Yes | Yes | Yes | Yes | Yes | Yes | Yes | Yes |
| Hulley (2016) | Yes | Yes | Yes | Cannot tell | Yes | Yes | Yes | Cannot tell | Yes |
| Kewley et al. (2017) | Yes | Yes | Yes | Yes | Yes | Yes | Yes | Cannot tell | Yes |
| Kitson-Boyce et al. (2019) | Yes | Yes | Yes | Yes | Yes | Yes | Yes | No | Yes |
| Kras (2021) | Yes | Yes | Yes | Yes | Yes | Yes | Yes | No | Yes |
| Kras & Blasko (2016) | Yes | Yes | Yes | Yes | Yes | Yes | Yes | No | Yes |
| Kruttschnitt, et al. (2000) | Yes | Yes | Yes | Cannot tell | Yes | Yes | Yes | Cannot tell | Yes |
| Lytle et al. (2017) | Yes | Yes | Yes | Yes | Yes | Yes | Yes | Yes | Yes |
| N. Mann et al. (2019) | Yes | Yes | Yes | Yes | Yes | Yes | Yes | No | Yes |
| McAlinden et al. (2017) | Yes | Yes | Yes | Cannot tell | Yes | Yes | Yes | No | Yes |
| Milner (2017) | Yes | Yes | Yes | Yes | Yes | Yes | Yes | Yes | Yes |
| Richards (2020) | Yes | Yes | Yes | Yes | Yes | Yes | Yes | Cannot tell | Yes |
| Richards et al. (2020) | Yes | Yes | Yes | Yes | Yes | Yes | Yes | Yes | Yes |
| Sample et al. (2018) | Yes | Yes | Yes | Cannot tell | Yes | Yes | Yes | Yes | Yes |
| Scoones et al. (2012) | Yes | Yes | Yes | Yes | Yes | Yes | Yes | Yes | Yes |
| Sowden & Olver (2017) | Yes | Yes | Yes | Yes | Yes | Yes | Yes | Yes | Yes |
| Stansfield et al. (2019) | Yes | Yes | Yes | Yes | Yes | Yes | Yes | Yes | Yes |
| ten Bensel, & Sample (2017) | Yes | Yes | Yes | Yes | Yes | Yes | Yes | Yes | Yes |
| ten Bensel & Sample (2019) | Yes | Yes | Yes | Yes | Yes | Yes | Yes | No | Yes |
| Walker et al. (2017) | Yes | Yes | Yes | Cannot tell | Yes | Yes | Yes | Cannot tell | Yes |
| Willis & Grace (2008) | Yes | Yes | Yes | Yes | Yes | Yes | Yes | Cannot tell | Yes |
| Willis & Grace (2009) | Yes | Yes | Yes | Yes | Yes | Yes | Yes | Cannot tell | Yes |
| Wilson et al. (2009) | Yes | Yes | Yes | Yes | Yes | Yes | Yes | No | Yes |
| Wilson et al. (2007) | Yes | Yes | Yes | Yes | Yes | Yes | Yes | Yes | Yes |
| Woodward (2018) | Yes | Yes | Yes | Cannot tell | Yes | Yes | Yes | No | Yes |

*Note.* Adapted from Critical Appraisal Skills Programme (2019a, 2019c, 2019d, 2019e)

Supplementary Material Table 6
*Identified Protective Mechanisms and Contributing Studies (N* = 29)

| Study | Prosocial Response to Formal Social Control | Positive Treatment Experiences | Development of Insight and Application of Understanding | Strengthened Personal Agency | Social Support and Accountability to Social Networks | Living In Congruence with Values | Desistance By Deterrence | Natural Desistance |
| --- | --- | --- | --- | --- | --- | --- | --- | --- |
| Bartle (2012) | x | x | x | x | x | x | x | x |
| Bates et al. (2012) | x | x | x | x | x |  |  |  |
| Bates et al. (2014) | x | x |  |  | x | x |  |  |
| Bohmert et al. (2018) | x | x | x | x | x |  |  |  |
| Cesaroni (2001) | x |  |  |  | x |  |  |  |
| Cooley (2020) |  | x | x | x | x | x | x |  |
| Cooley et al. (2017) | x | x | x | x | x | x | x |  |
| Cooley and Sample (2018) | x | x | x | x | x | x | x |  |
| Duwe and Donnay (2008) | x |  |  |  |  |  |  |  |
| Farmer et al. (2012) |  | x | x | x | x | x |  |  |
| Farmer et al. (2015) | x | x | x | x | x | x | x |  |
| Farmer et al. (2016) |  | x | x |  |  | x | x |  |
| Harris (2014) |  | x | x | x | x | x | x | x |
| Harris (2016) | x | x | x | x | x | x | x | x |
| Harris (2017a) |  | x | x | x | x | x | x | x |
| Harris et al. (2017) | x |  | x | x | x | x |  |  |
| Harris et al. (2019) | x | x | x | x | x | x |  |  |
| Harris and Levenson (2021) | x | x | x | x | x |  | x |  |
| Höing et al. (2013) |  | x | x | x | x | x |  |  |
| Höing et al. (2017) | x | x | x | x | x | x |  |  |
| Hulley (2016) |  | x | x | x | x | x |  |  |
| Kewley et al. (2017) |  | x | x |  | x | x |  |  |
| Kitson-Boyce et al. (2019) |  | x | x | x | x |  |  |  |
| Kras (2021) |  | x | x | x | x | x | x |  |
| Kras and Blasko (2016) |  |  | x |  |  |  |  |  |
| Kruttschnitt et al. (2000) | x |  |  | x | x | x |  | x |
| Lytle et al. (2017) |  | x |  |  | x | x | x |  |
| N. Mann et al. (2019) |  | x | x | x | x | x | x |  |
| McAlinden et al. (2017) |  | x | x | x | x | x | x | x |
| Milner (2017) | x | x | x | x | x | x | x | x |
| Richards (2020) |  | x | x |  | x | x |  |  |
| Richards et al. (2020) |  | x | x | x |  | x | x |  |
| Sample et al. (2018) |  | x | x | x | x | x | x |  |
| Scoones et al. (2012) |  | x |  |  | x |  |  |  |
| Sowden and Olver (2017) | x |  |  | x |  |  |  |  |
| Stansfield et al. (2019) |  |  |  |  | x |  |  |  |
| ten Bensel and Sample (2017) |  | x | x | x | x | x |  |  |
| ten Bensel and Sample (2019) |  | x | x |  | x | x | x |  |
| Walker et al. (2017) |  |  |  |  | x |  |  | x |
| Willis and Grace (2008) |  | x |  |  | x |  |  |  |
| Willis and Grace (2009) | x | x |  |  | x |  |  |  |
| Wilson et al. (2009) | x |  |  |  | x | x |  |  |
| Wilson et al. (2007) | x |  |  |  |  |  |  |  |
| Woodward (2018) | x | x | x | x | x | x | x |  |
| Studies per mechanism | *n =* 16 | *n =* 21 | *n =* 18 | *n =* 17 | *n =* 26 | *n =* 18 | *n =* 11 | *n =* 6 |

*Note. N* = 44 reports informed *n* = 29 studies.

Supplementary Material Table 7

*Examples of Findings that Informed Each Mechanism and Key Themes*

| Mechanism and Theme | Examples of Findings |
| --- | --- |
| *Prosocial Response to Formal Social Control* | |
| Turning points for change | “I remember the day that they opened that gate and let me out, and it was just like, I’m never going to go back there again” (Bartle, 2012, p. 77).  “Interviewees repeatedly said that they were ‘shocked’ into changing not just their behaviours but also their views about the abuse they were perpetrating, and this precipitated an end to any consideration of further abuse. A number of participants vividly described their shock at being arrested. Several said that arrest acted as a turning point after which they ceased offending” (Farmer et al., 2015, p. 328).  “The shock of the police turning up at the door- gave me enough of a shock not to want to do it again” (Milner, 2017, p. 81). |
| Active engagement with professionals | “I wanted to fulfil the terms of the probation. I wanted to have my life back. I wanted to, like you hear over and over again, I wanted to be that changed person. I also, as time went on, felt better about myself for doing as well as I was doing, working hard in the therapy sessions. The first two years, I got a lot out of therapy. I even started to collaborate with my probation officer” (Cooley et al., 2017, p. 127).  “I just started going to church again. But I had to go through some stuff to make sure I could go because I can’t be around kids and stuff like that” (Harris et al., 2017, p. 112).  “His sense of self was changing because he was able to prove to others that he was no longer a risk and he was happy to engage with all of the requirements of his risk management plans” (Woodward, 2018, p. 219). |
| Positive Treatment Experiences | |
| A turning point for change | “I know that part of it has to be therapy. That’s why I guess they say knowledge is power. See, I wasn’t armed with all the information before, so I wasn’t reasoning as well as I am now” (Harris, 2014, p. 1567).  “Desisters discussed how they were able to assert agency over their lives because treatment lessons supported overall life changes, not just criminogenic ones” (Kras, 2021, p. 13).  “Participants about their turning point in relation to deciding to stop offending. The men gave four key reasons (in order of prevalence): Effect on self and others, Treatment, Arrest and prison and Wanting a positive future” (Milner, 2017, p. 100). |
| Peer support and mutual challenge | “After two months of sobriety his confidence in the process had grown, and the Circle began to challenge continuing poor attitudes towards women… His willingness to discuss live issues in this way showed promise in the process (Bates et al., 2012, pp. 366–367).  “It gives me a place to talk about it. It gives me a place to vent. I can see that there’s other people having problems like I do. That helps a lot, the fact that you know that there are other guys who are going through the same kind of stuff you are.” (Kras, 2021, p. 14)  “Marvin (registrant) explained, “I could have not gotten through my conviction and sentence without this group. So much encouragement here” (Sample et al., 2018, p. 4268). |
| Understanding sexual offense process | “Once you understand what you’ve done wrong, you can start moving on to how to counteract that so you don’t do it again” (Bartle, 2012, p. 90).  “During the interviews, they were eager to demonstrate their newly gained insight regarding offending and self-governance. They willingly shared their knowledge of triggers, tools, and risk situations and spoke in the present tense about their ability and commitment to stay safe” (Harris, 2016, p. 1728).  “Basically the programme properly sorted me out… I learnt coping techniques and talking about things without trivializing it or putting it on them [victims]… I learnt the skills to see the warning signs and I now know who I’ve got to talk to” (N. Mann et al., 2019, p. 6). |
| Awareness of the harm caused to others | “Acknowledging the harm caused by their offense emerged as an important theme in the narratives of the men. When asked to identify a general turning point in their lives, many participants spoke of the (extremely difficult) moment that they accepted what they had done and took responsibility for their offense” (D.A. Harris, 2014, p. 1567).  “Desisters’ redemption occurred as a process of developing empathy, which improved agency and self-efficacy, ultimately impacting self-worth…for instance, when asked about how he knows he will not reoffend again, Louis stated, “The principles, I utilize the principles. I try to put myself in somebody else’s shoes. I want to treat people like I want to be treated.” In essence, the treatment experience changed desisters’ views of themselves such that engaging in sexual deviance was incompatible with their emerging identity and developing empathy” (Kras, 2021, pp. 12-13).  “Definitely the SOTP had the biggest effect on stopping me from reoffending. Because of the victim empathy, that’s when I decided” (Milner, 2017, p. 81). |
| Development of Insight and Application of Understanding | |
| Self-regulation | “Changes included coping better with emotion, reducing stress, avoiding drug and alcohol use, and making sure that basic life requirements were in place… Participants described getting better at handling strong emotions including anger, resentment and sadness, and also spoke about having a better understanding of their emotions, which led to more effective coping strategies” (Bartle, 2012, pp. 107–108).  “That’s when I first learned to meditate and was able to get some separation from my emotions and get a way to subjectively look at my emotions from outside. . . . And that has just been an incredible journey. . . . So, every day now is about, I think, my life right now. You know? Every moment of my day right now is a high point, no matter what I’m dealing with because I know that it’s me living it now” (Harris et al., 2019, p. 210). |
| Relationships | “Registrants discussed how although they were married to the same woman before their sexual crime, their relationship was stronger because they communicated better and were able to share anything with their spouse” (Cooley, 2021, p. 59).  “Some core members learned to be more open and honest in their communication, and some report an improved quality of their relationships outside of the circle, due to more openness in their communication” (Höing et al., 2013, p. 280).  “There’s just me (name) now, but old (name) wasn’t very nice, he was a predator, terrible, where new (name) is open, honest with people and treats people with respect, and they treat me with the respect I have never had" (Milner, 2017, p. 79). |
| Safety | “The Circle worked with John to help him manage risky situations. A good example is the use of a checklist they helped him to produce. It is laminated and on the inside of his front door. It gives him a set of responses to say if any neighbours knock at his door with requests that might place him in risky situations” (Bates et al., 2012, p. 365).  “I can go to church if I get an ‘okay’ from the pastor or minister or whatever. If I get an ‘okay’ from them that I told them my charges, and they’re gonna put me in a seat where there ain’t no young children around or nothing like that” (Harris et al., 2017, p. 112). |
| Shame management | “Situational aspect of their offending past is a narrative frame that the individuals have retrospectively applied to past behaviours so as to separate their desired, present selves from undesirable past selves. In such cases, the framing itself might be understood as a protective cognition that helps them desist” (Farmer et al., 2015, p. 327).  “Still others, overcome by the stigma of their offense and now understanding the devastating consequences of their crimes, may decide quite rationally to never offend again, live an offense-free life, avoid another stint in prison, or the creation of more victims.” (Harris, 2014, p. 1575).  “Neutralizations assist desistance from sexual offending by allowing for the negotiation of stigma and rejection of the “sex offender” label, thus providing for the development of a nonoffending, prosocial identity” (Hulley, 2016, p. 1776). |
| Strengthened Personal Agency | |
| Internal locus of control | “Through Agency they were able to have a sense of control, which enabled them to feel that they could exercise some command over what happened in their life, in that they were able to find positive outcomes from negative events” (Farmer et al, 2012, p. 942).  “It’s up to us [to not reoﬀend]”, that “keeping out of trouble is up to you” and that “it’s your choice to walk the road”. Men in the study did not identify an external savior (e.g., program or person) responsible for recognizing their true potential, but rather took credit for their own desistance” (Richards et al., 2020, p.16). |
| Self-efficacy | “For many of the men, informal social controls, such as family, were a positive aspect of their lives, encouraging them to remain offense free: I’m very optimistic I’ve got family… it gives me a feeling of self-belief and self-esteem” (N. Mann et al., 2019, p. 9).  “The men also took ﬁerce ownership of their current and future law-abiding selves, displaying a sense of optimism about the future and a strong sense of self-eﬃcacy” (Richards et al., 2020, p. 16). |
| Self-esteem and confidence | “In addition, core members report a more positive mental self-representation (self-esteem, positive narrative identity). “I feel more self conﬁdent, have more trust in the future. My fears that I don’t belong in this society anymore have gone. I do belong” (Höing et al., 2013, pp. 280-281).  “Group members reported increased levels of self-esteem, confidence, empowerment, and changes in the way they see themselves since becoming members of “Fearless.” Huck explained how he would not have the confidence to testify at legislative hearings without the encouragement of “Fearless” members” (Sample et al., 2018, p. 4268). |
| Perseverance and resilience | “The final style of desistance is “resilience” and includes a constellation of characteristics that emphasize recovery and redemption. This final style was marked by a strong desire to move on from their offending past as well as emphasizing a demonstrated pattern of success upon release” (Harris 2016, p. 1729).  “Even though this resettlement process was slow and it had taken eight years for him to feel more settled than he ever had, he stated he was determined and motivated to succeed with his life away from crime” (Woodward, 2018, pp. 136–137). |
| Hope and optimism | “Resilient desisters tended to look to the future with hope and optimism and spoke convincingly about their recovery and rehabilitation” (Harris, 2016, p. 1724).  “This research supports previous (mainly) theoretical suggestions that hope and optimism, locus of control and social connectedness may be important factors in desistance from sexual offending” (Milner, 2017, p. 165). |
| Social Support and Accountability to Social Networks | |
| Acquisition of social support | “Social support and romantic partners were named as particularly important in terms of mitigating the desistance-undermining effects of negative consequences, but also in assisting with risk awareness and management, reminding participants of prosocial strengths and values, and providing opportunities to practice and benefit from positive changes” (Bartle, 2012, p. 120).  “The passage of time and acquisition of social capital appeared to provide respondents desisting from sexual offending with meaning in life as they engaged in the process of identity reconstruction” (Hulley, 2016, p. 1786).  ““There’s not a lot you can’t get through even if it’s something you can’t singularly (get though), you can get together with someone (from the circle).” (Kitson-Boyce, 2019, p. 201) |
| Support and accountability | “Nearly all the Core Members interviewed stated that having the volunteers to talk to helped them work through problems and respond to them in a risk-free way” (Kitson-Boyce et al., 2019, p. 200).  “I’ve had very supportive friends and people that are around me. My kids are very supportive as well. They’ve been there for me. When I sat there fighting with depression… family really kept me, got me back straight again. That’s the most important thing” (N. Mann et al., 2019, p. 9). |
| Prosocial roles | “Over time, Jack has come to see himself as a productive, working citizen with hopes and dreams that model those of other prosocial others. He no longer looks to his feet when he speaks, avoids shaking hands, or appears tense during interviews. He is now relaxed, calm, and freely shares changes in his life circumstances and how they make him feel. He is proud of himself for who he is now, a pride not observed when interviewing him as he reentered society and began living without any correctional supervision” (Cooley et al., 2018, p. 493).  “These friendships and group activities helped empower sex offenders not only to adopt, but to maintain a conventional prosocial identity that may help them desist from offending” (ten Bensel & Sample, 2017., p. 506). |
| Prosocial connection | “Regardless of the context, country, system, or structure in which Circles are implemented, it would appear that the “magic ingredient” of meaningful human relationships remains the same. It works because members of the community give of their time and selves through an apparently genuine desire to engage in constructive and creative ways of reducing sexual reoffending” (Bates et al., 2014, p. 879).  “Supportive relationships provided a source of uniformity and acceptance for desisters, which is important considering the ostracizing stigma typically experienced by sex offender populations” (Lytle et al., 2013, p. 132). |
| Living in Congruence with Values |  |
| Living a meaningful life | “I have a life I want, and I have done things to get that. I got help, I have friends so I don’t have to deal with everything alone, and my job is great” (Cooley & Sample, 2018, p. 492).  “I know where I’m going, I know what I want to do. I’ve got a circle of friends now, my parents are aware of me being bullied (as a child), I’m back home with them now, my money’s under control, my drinking is under control, the only thing that isn’t under control . . . is the sickness because I had five weeks off work this year with it but now I’m onto medication finally to control it” (Farmer et al, 2012, p. 941).  “Majority of core members in our sample showed—to a varying degree—signs of transitions toward desistance. Most prevalent were changes in cognitive function, for example, improvements in self-reflection, self-confidence, and self-esteem; and behavioral changes, such as more active problem solving, improved assertiveness, and improved social skills” (Höing et al., 2017, p. 767). |
| Sense of purpose | “I feel a lot better with my life being able to help others due to being a volunteer and giving something back to the community” (Milner, 2017, p. 79).  “They all seemed to have found people they consider as friends, a sense of purpose, and little sense of social exclusion when actively participating in multiple online media formats” (ten Bensel & Sample, 2019, p. 287).  “Nearly all of the desisting group participants in the study portrayed themselves as being in control of their lives and had a clear sense of purpose and planning for the future” (McAlinden et al., 2017, p. 277). |
| Stability | “Two core members developed more appropriate leisure time activities to reduce the risk of relapse (“It helped me to talk about my risk factors—one of the things we discovered was the fact that I have little activities in the evenings and the weekends, which resulted in a list of things I can do and places to go—which I do now”)” (Höing et al., 2017, p. 762).  “One common sub-theme was the self-awareness of the importance of ‘keeping busy’ or remaining occupied as a situational coping mechanism. This seemed to be particularly the case for men who had offended over the internet in the past” (McAlinden et al., 2017, p. 272). |
| Spirituality and faith | “The opportunity to seek forgiveness was one that participants took during their engagement with their religious community. In navigating the forgiveness-seeking process, participants gained an immense sense of peace, restoration and comfort” (Kewley et al., 2017, p. 93).  “Sean had developed a relationship with his faith, as he proclaimed to be a Buddhist… he stated it helped him to live day-to-day and to enjoy the moment, rather than fix himself on goal setting. Buddhism gave Sean the peace of mind that he longed for and it helped him to feel part of a small community. It was non-judgemental in nature and that drew him to it” (Woodward, 2018, p. 152). |
| Prosocial identity | “The resilient desisters also spoke of having had two lives and of “knifing off” (Maruna, 2001). Akin to Giordano et al.’s (2002) “complete desisters,” these men tended to describe their offending in the past tense, thus placing a “great deal of distance between their old, discarded selves and those they currently claim” (p. 1031)” (Harris, 2016, pp. 1729–1730).  “The men in the study were therefore all “primary desisters” in that they had not reoﬀended following their release from prison. In the main they also presented as “secondary desisters”– i.e. presented identity narratives incompatible with oﬀending” (Richards et al., 2020, p. 11).  “This capacity to embrace a new non-criminal identity and to move on from the legacy of an offending past or to ‘put the past behind them’ was integral to the desisting narratives of the participants in the present study” (McAlinden et al., 2017, p. 276).  “Hence, for the (desisting) men who discussed it, twice as many described cognitive transformation as those still identifying as sexual offenders. Cognitive transformation primarily occurred through treatment. This research proposes that cognitive transformation is central to being a ‘Thriver’; that is, an individual with a set of other protective factors, such as hope and optimism, social connectedness and an ability to manage one’s sexual interests through cognitive strategies” (Milner, 2017, p. 209).  “Upon interviewing, Kevin explained he prefers to be called a ‘registered citizen’ rather than a ‘sex oﬀender’ and he clearly associates himself with the legal status of sex oﬀender, not a personal identity of a sex oﬀender” (Cooley et al., 2018, p. 495).  “The label “sex offender” carries considerable negative connotations and was openly rejected by many of the participants in this study. In this way, participants may have been considering the impact of such labelling on their future selves, and attempting to construct a more positive personal identity. They did not wish to be viewed as a “sex offender,” and they did not wish others to view them in this way either” (Farmer et al., 2016, p. 1767). |
| Desistance by Deterrence | “Resigned desisters were stuck in the past and used a lot of negative language, often in the past tense… None of these men identified themselves as sex offenders and expressed frustration that they were required to participate in treatment for something that they often described as a “oneoff,” blamed on substance abuse, and/or maintained was part of a consensual relationship” (Harris, 2016, p. 1724).  “Most participants displayed a style of coping which was accented by crippling and ultimately futile hypervigilance…obsessive about complying with the law to avoid exposure or re-incarceration” (Harris & Levenson, 2021, p. 776).  “Men who still identified with being a sex offender. They had not undergone a cognitive transformation, and the clinical observation was that they appeared more socially isolated, less hopeful and had a Poor me victim stance. Their turning points for change were external; the impact of arrest and prison. This group was not offending, yet appeared to be just ‘surviving’ and existing day to day” (Milner, 2017, pp. 173–174). |
| Natural Desistance | “The three men identified as “natural desisters” were among the youngest in the sample (aged 44-48). The men who talked explicitly about aging out of crime did so in very typically criminological ways…”I’m not like I used to be. I don’t get violent. I don’t drink and do drugs today. (Subject 10) It’s just not in me no more. I’m too old . . . I got tired of fighting, y’know?”” (Harris, 2014, p. 1566).  “I’m just getting past it, I am too old.” (Milner, 2017, p. 97)  “Offending rates declined with time for everyone in the sample, irrespective of the level of family support. This finding is unsurprising, given the overwhelming evidence that crime tends to decline with age, both for individuals convicted of general offenses and for those convicted of sex offenses” (Walker et al., 2017, p. 16). |
